# Mood modulations of emotional word processing: A predictive perspective view of EEG data

**DOI:** 10.3758/s13415-025-01394-x

**Published:** 2026-03-25

**Authors:** Ekaterina Kopaeva, Johan Blomberg, Mikael Roll

**Affiliations:** Center for Languages and Literature, Lund University, PO Box 201, 22100 Lund, Sweden

**Keywords:** Mood, Affective state, Emotional language processing, Event-related potential, Language, Semantics

## Abstract

Mood is a pervasive affective state that influences cognition, including language processing, where it functions as part of the pragmatic context. When linguistic expressions carry emotional valence, mood may amplify or attenuate their perceived emotional nature. Motivated by inconsistent findings on the timing and nature of mood and valence interactions, we conducted an EEG study to investigate whether induced moods alter the temporal profile of emotional word processing. Participants rated words of high, low, and neutral valence in a control condition and after positive and negative mood induction. Event-related potentials were analysed across three early processing windows using cluster-based permutation tests. Results showed that mood modulated early neural activity: in the positive mood, high-valence words reduced N1 amplitudes compared with control, reflecting diminished prediction error. In the subsequent P2 and EPN windows, reduced amplitudes for high- and neutral-valence words indicated a decreased need for model updating under mood-congruent expectations. By contrast, the negative mood exerted weaker and later effects. Behavioural responses corroborated these findings, with faster response times to valenced words. Within predictive coding frameworks, mood can be understood as a hyperprior that shapes precision weighting and error dynamics. Our results suggest that induced positive mood facilitates the processing of congruent words and broadens the range of input treated as predictable, whereas induced negative mood primarily reduces positive affect. These findings highlight the role of mood in dynamically tuning early language processing through the regulation of prediction error and model update.

**Supplementary Information:**

The online version contains supplementary material available at 10.3758/s13415-025-01394-x.

## Introduction

Mood has long been recognised as a factor shaping cognitive functions, such as perception, attention, memory, and evaluation (Fiedler, 2001; Forgas, 1995). In particular, mood can bias the way affect is attributed to stimuli: in a positive or negative affective state, neutral objects or events are often evaluated in line with the individual’s current mood (Schwarz & Clore, 1983). When stimuli already carry affective valence, the interaction becomes more complex, as mood may either amplify or attenuate the evaluative response. This raises the question of how the comprehender’s mood and stimulus valence combine during real-time processing, especially in a domain as rapid and complex as language. In language comprehension, valence effects have been reliably observed in electrophysiological measures, beginning around 200 ms after word onset (Kotz & Paulmann, 2011). Yet whether mood modulates these early stages of valence processing remains unresolved. Recent predictive models of mood (Clark et al., 2018; Kiverstein et al., 2020) propose that mood operates as a higher-order constraint that regulates the balance between predictions and sensory evidence, thereby shaping which input is treated as reliable. Building on this perspective, we focused on the early time window of visual word processing and used event-related potentials (ERPs) to investigate how mood interacts with valence at these initial stages to capture the temporal dynamics of this interaction.

### Mood and cognition

An individual’s emotional state, or mood, has been shown to influence cognitive processes, such as perception, memory, attention, and evaluation (Fiedler, 2001). Understood as a diffuse, relatively slow-changing affective state, mood is characterised by low intensity, is less nuanced than emotion, and conveys rough information about the valence of one’s experience (Forgas, 1995; Frijda, 2009). Rather than being a response to a particular stimulus, like emotions are, moods may lack a single cause, and the underlying reason for experiencing a particular mood is not always consciously available (Damasio, 1996; Forgas, 1995). Consequently, mood can result in affect misattribution, when the perceived valence of an external stimulus is evaluated in line with the individual’s current affective orientation (Schwarz & Clore, 1983). Studies of the effects of mood on processing have gone from suggesting a straightforward mood-congruent effect (Bower, 1981) to more nuanced accounts, which consider the factor of task (Forgas, 1995), biological motivations associated with moods (Fiedler, 2001), and adaptive functions of mood (Schwarz, 1990).

Effects of mood on cognition are commonly attributed to varying information-processing styles that different moods impose (Forgas, 1995). Mood biases an individual towards either reliance on heuristics or on sensory input. Specifically, positive mood is characterised by reliance on prior experience and knowledge of the world and by exploratory behaviour, quicker attention capture, and faster disengagement. Negative mood is associated with a processing style promoting hypervigilance, avoidant behaviour, and greater attention to detail (Fiedler, 2001; Forgas, 1995; Schwarz & Clore, 1983; Schwarz, 1990). In essence, these classical sociocognitive models suggest that positive mood relies more on top-down processing and is driven by schemas, while negative mood promotes bottom-up analysis and relies on sensory input.

Recently, new models of mood have started to emerge in cognitive neuroscience. These models, rooted in prediction, aim to account for the effects of mood by regarding it as a system-wide parameter that regulates how strongly predictive models and sensory input influence processing. Both approaches converge on the idea that mood regulates the relative influence of top-down knowledge and bottom-up information in shaping processing. However, while classical sociocognitive models describe this shift primarily at the level of cognitive strategies and observable behaviour, predictive accounts aim to explain it in terms of underlying neural computations.

### Prediction and mood

Predictive coding proposes that cognitive, perceptual, and motor activity arises from continuously comparing internally generated predictions with incoming sensory input, updating the internal model to minimise prediction error (Clark, 2013; Friston, 2009). Sensory signals provide information about the external world, which the brain interprets through active inference based on prior experience. Discrepancies between predictions and input generate prediction errors, which propagate hierarchically to higher processing levels to refine the model. Not all prediction errors are treated equally; their influence depends on precision, reflecting the reliability of the sensory input and modulated by attention or sensory gain. Highly precise priors resist change, whereas flexible priors can be updated by even low-precision input (Friston, 2009; 2018).

Predictive coding extends beyond exteroception to interoception, providing a framework for understanding affective phenomena, such as emotion and mood (Barrett, 2017; Clark et al., 2018; Kiverstein et al., 2020; Kube et al., 2020; Seth, 2013; Seth & Friston, 2016). In both domains, prediction errors can be minimised in three ways: updating internal models, altering sensory input via action, or adjusting the precision of sensory signals through attention or attenuation (Clark et al., 2018; Friston, 2009). In the frameworks recently developed in computational neuroscience (Clark et al., 2018), psychology (Kiverstein et al., 2020), and interoceptive models of depression (Kube et al., 2020), mood is viewed as a higher-order construct that aims to minimise free energy through these interventions.

In these frameworks, mood is understood as a regulator of prediction and precision-weighting that sets a baseline for how the system interprets input. It establishes a set-point that determines which input is anticipated, which predictions carry high weight, and which input generates the most prediction gain. Clark et al. (2018) define mood as a hyperprior that shapes expectations about uncertainty, determining the strength of prior beliefs and the model’s sensitivity to error. Kiverstein et al. (2020) emphasise the momentum of error dynamics, where mood reflects the expected trajectory of free-energy reduction. Together, these accounts converge on the idea that a healthy affective state is characterised by a model that is moderately precise and open to update. In a positive mood, the model assumes that the world is controllable and uncertainty resolvable. Prediction errors consistent with the expectation are assigned a high precision weight and thereby strengthen the model. By contrast, prediction errors that signal threat or instability are granted low prediction gain and have less impact. In a negative mood, the model assumes, with a high degree of certainty, that the environment is volatile, unpredictable, and uncontrollable. Incoming signals that confirm unpredictability are given high precision weight (get attention), while counterevidence to the model is given low prediction gain (is attenuated). Across all states, the model selectively suppresses the input that is not consistent with it.

In sum, predictive frameworks complement well-established sociocognitive views on mood as a regulator of cognition that tilts the balance between top-down and bottom-up processing. In predictive models, mood is treated as a hyperprior (Clark et al., 2018) or a background affective orientation (Kiverstein et al., 2020) that shapes which input is attended to, based on how predictable or volatile the world appears. In a positive mood, the world is assumed to be safe and controllable, meaning that a wide range of incoming signals can be seen as consistent with the model. As a result, more signals are treated as reliable and precision-weighted. By contrast, a negative mood sets the expectation that the world is uncertain and unsafe, therefore narrowing attention to threat-consistent evidence and attenuating other types of signals. In both cases, input that contradicts the belief is down-weighted, while congruent input is amplified. It is the scope of what falls into the category of model-congruent input that makes processing appear more top-down or bottom-up.

### Prediction and language processing

Prediction in language comprehension has mostly been studied using paradigms that focus on the two phases of target processing (León-Cabrera et al., 2024). During the pre-target processing stage, activity at a predictive cue is measured. The predictive cue may take the form of a lexical tone (Roll et al., 2015), morpheme, word, or word segments (Roll et al., 2017), and it restricts the model in its choice of plausible options for the target. Slow pre-activation negativities mark the trigger: the more restrictive the trigger, the greater the elicited amplitude. During post-target processing, there is a response depending on whether the prediction was matched or mismatched by the target.

The function of the cue is to pre-activate information. That is to say, the lexical tone, morpheme or word creates a context in which some continuations are more plausible than others. *Context* is operationalized as the preceding words or word segments and their semantic or syntactic properties. However, when predicting, individuals consider other types of contextual information which is not strictly linguistic, such as the broader pragmatic context and world knowledge (Ryskin & Nieuwland, 2023). In line with the Affective Comprehension Model (Van Berkum, 2018), the emotional state of the recipient is an important factor in shaping the semantic content of the perceived message, and mood has been regarded as such a context in earlier works, including on decontextualised words (Kissler & Bromberek-Dyzman, 2021).

Predictive perspectives on mood have begun to permeate experimental research in psychology, particularly in the study of affective disorders (review in Kube et al., 2020; Gilbert et al., 2022). A series of paradigms now examine how mood biases the balance between predictions and sensory evidence or alters the weighting of prediction errors. For instance, Rutledge et al. (2014) showed that fluctuations in momentary happiness during a probabilistic reward task were best explained not by absolute gains, but by recent reward expectations and their associated prediction errors, highlighting mood’s predictive structure. Whitton et al. (2015) demonstrated blunted neural responses to reward prediction errors in depression, while Browning et al. (2015) found that anxious individuals overestimated environmental volatility, which results in maladaptive belief update. Together, these findings suggest that mood operates as a contextual factor shaping how predictions are generated and updated. Experimental research, as well as the theoretical frameworks outlined above, suggest that mood can be understood as a higher-order constraint that tunes the system toward certain kinds of expected input. It is therefore important to consider mood a potentially decisive factor in predictive processing in language comprehension as well.

### Mood and language processing

When effects of mood on information processing, including in language, are investigated in nonclinical populations, mood induction is performed—a controlled manipulation of a person’s affective state for a limited amount of time. Participants’ performance in positive and negative induced states is then compared against each other or a control.

In studies of language processing, effects of induced moods have mostly been reserved for the positive mood induction (Kiefer et al., 2007; Kissler & Bromberek-Dyzman, 2021; Naranowicz et al., 2022a; 2022b; Pratt & Kelly, 2008; Sereno et al., 2015; Van Berkum et al., 2013; but see Egidi & Nusbaum, 2012). Induced positive mood tends to facilitate the processing of congruent verbal targets (Egidi & Nusbaum, 2012; Kiefer et al., 2007; Sereno et al., 2015, but see Pratt & Kelly, 2008), and valence differentiation has mostly been observed in a positive mood only (Kiefer et al., 2007; Naranowicz et al., 2022a; Pratt & Kelly, 2008; Sereno et al., 2015; review in Naranowicz, 2022). This may come as a surprise, considering that the emotional content of a word has been reliably shown to facilitate semantic processing when mood is not accounted for (Blomberg et al., 2020).

The interaction of mood and valence in word processing was studied by Naranowicz et al. (2022a), who found facilitation for neutral and positive words in a positive induced mood, and a mood-congruent facilitation in a positive mood, but only among female participants. Sereno et al. (2015) observed mood congruence in a positive mood in a lexical decision task, where positive words lost their initial advantage post mood induction. The pattern was not replicated in a negative mood. The results were explained as an effect of mood on executive attention, whereby a positive mood broadens attention, thus erasing the differences among words of different valence. In an ERP study, Kissler & Bromberek-Dyzman (2021) had bilinguals perform a semantic categorisation task after being put in a positive and negative mood. The authors reported increased N1 amplitudes for congruent targets over parieto-occipital regions in a positive mood, although they expected effects in both moods. With no control mood condition, however, it is hard to say whether the observed valence contrast is the result of an emerging effect in a positive mood or its attenuation in a negative mood. The problem of a lack of a control group has been seen as a drawback and a hindrance in analysing found effects (Cheng et al., 2017; Chwilla et al., 2011).

Another problem is determining when an interaction of mood and valence occurs. Mood is a preexisting internal state of a subject; hence, mood effects should have an early onset and start at least as early as the processing of the word form. However, it is up for debate when emotional features of a stimulus are accessed.

### Serial and parallel processing in language

Models of serial processing assume that the analysis of sensory features of a stimulus, lexical identification, and contextual integration form a sequence of distinct stages. For written language, visual form analysis occurs in the first 200 ms after the word onset, after which semantic representations are accessed, and contextual integration takes place at around 400 ms, followed by syntactic integration at around 600 ms (review in Hinojosa et al., 2020; Kotz & Paulmann, 2011). Proponents of parallel processing models expand these ideas by underlining that only later processes, which occur after access to semantic representations, follow well-defined stages (Pulvermüller et al., 2009). Within the first 200 ms after word presentation during reading, top-down and bottom-up processing may occur nearly simultaneously. That is, information about the physical features of the word (Schindler et al., 2018; Simon et al., 2007), lexical (Palazova et al., 2011; Scott et al., 2009), and semantic, grammatical, and syntactic features (review in Pulvermüller et al., 2009) are all processed rapidly within the first 200 ms after the word presentation. Recent studies of emotional word perception find evidence supporting models of early parallel processing (Herbert, 2022; Kissler & Bromberek-Dyzman, 2021), finding emotion-related effects as early as 100 ms after word presentation, although these reports lack consistency.

#### P1

The P1 is a distinct positive peak at around 100 ms at occipital sites that is thought to register early sensory properties of visual stimuli (Luck & Kappenman, 2012). Its source is localised in the extrastriate cortex responsible for early visual perception (Luck & Kappenman, 2012) and the middle temporal gyrus (MTG) and fusiform gyrus, which are also involved in lexico-semantic processing (Hinojosa et al., 2020). The last two areas may account for P1’s differential response to emotional words as opposed to neutral ones. Some studies have shown that emotional words elicit higher P1 amplitudes than neutral ones (review in Citron, 2012; Kissler et al., 2006). Some also found a negativity bias in this time window (explained by the automatic vigilance hypothesis (Pratto & John, 1991)), with higher amplitudes elicited by negative words as opposed to positive and neutral ones (Sass et al., 2010; Zhang et al., 2014). Other studies have failed to find such early effects of emotion (Herbert et al., 2008; Herbert, 2022; Kissler et al., 2009; Pauligk et al., 2019; Zhang et al., 2017). Several studies (Palazova et al., 2011; Scott et al., 2009) found no effect of emotion but did see an interaction of emotional valence of the word with its frequency. These mixed findings do not allow us to label the P1 as responsive to emotion only, yet they go to show that psycholinguistic properties of the stimuli are processed nearly simultaneously, supporting models of parallel processing.

#### N1

The N1 is treated as a marker of visual attention responsive to lexical properties of the word, such as frequency. The visual N1 has subcomponents and two loci of distribution: a frontal and a parietal one, of which the former is believed to have a slightly earlier onset (Luck & Kappenman, 2012). The N1 follows the P1 and, together with it, is believed to respond to modulations of exogenous factors (Luck & Kappenman, 2012). Regarding its response to emotional properties of a stimulus, research output is not homogenous either. In a number of studies, no effect of emotion was found this early (Herbert et al., 2008; Pauligk et al., 2019; Zhang et al., 2017). A few studies found that emotional words elicited more pronounced amplitudes than neutral ones (Jia et al., 2022; Kissler & Herbert, 2013; Sass et al., 2010; Zhang et al., 2014). Some studies reported a positivity (Herbert, 2022; Kissler & Bromberek-Dyzman, 2021) or negativity bias (Bernat et al., 2001 in Kissler et al., 2006; Kissler & Herbert, 2013) when processing emotional words. An interaction between semantic and psycholinguistic features (e.g., emotion and frequency, Scott et al., 2009) also support the idea of a near-simultaneous access to psycholinguistic information. Inconsistencies in the N1 literature may be due to a lack of clarity about the component label and distribution.

#### P2

Following the N1 is a positive peak at central anterior sites, approximately 180 ms after the onset of the stimulus, known as the P2 (Hajcak et al., 2012). Together, they form the N1-P2 complex. Increased P2 amplitudes have been observed in response to emotional as opposed to neutral words (Herbert et al., 2008; Kanske & Kotz, 2007; Kissler et al., 2006; Schapkin et al., 2000), and the component has been thought to indicate postperceptual selective attention to emotional stimuli (Hajcak et al., 2012; Kanske & Kotz, 2007).

#### EPN

Preferential processing of emotional language in visual presentation reliably starts at 200 ms post stimulus and is reflected in increased amplitudes elicited by emotional words in the window of the Early Posterior Negativity (EPN), a negative deflection at occipito-temporal sites between 200 and 300 ms (Hajcak et al., 2012), which is regarded as a marker of selective visual attention to specific stimulus features. Its activation is not only limited to linguistic stimuli, but also faces and images (Flaisch et al., 2008; review in Hajcak et al., 2012). It is linked to automatic attention allocation (Schupp et al., 2006) during implicit processing of emotion (Hinojosa et al., 2010; Kissler et al., 2006; Schacht & Sommer, 2009). The component is source-localised to the left extrastriate cortex and fusiform gyrus (Kissler et al., 2007; Schacht & Sommer, 2009). Language processing studies consistently report greater amplitudes for emotional rather than neutral stimuli between 200 and 300 ms (Herbert et al., 2008; Kissler et al., 2007; Kissler et al., 2009; Palazova et al., 2011). Negative words tend to elicit higher amplitudes than positive ones (Kiefer et al., 2007; Scott et al., 2009; Wu et al., 2021), although this is not consistent across all studies (Imbir et al., 2015; Kissler & Bromberek-Dyzman, 2021). It is therefore generally agreed that the component is arousal rather than valence specific.

### Summary and hypotheses

Considering all of the above, we investigated how early mood and valence interact during visual word presentation, and the nature of that interaction. Based on the literature, we expected to find valence effects at least starting from the first 200 ms after word presentation. Observing such effects prior to lexical access would be consistent with models of early parallel processing. From a predictive coding perspective, mood serves as a hyperprior that shapes how prediction errors are weighted. A positive mood establishes the expectation that the environment is safe and predictable, resulting in broadly distributed precision and thus a greater sensitivity to valence contrasts across words. A negative mood, in contrast, narrows precision onto negative-consistent inputs and down-weights incongruent evidence, producing a more selective processing style. In line with this, we expected valence differentiation to occur earlier and be more robust in a positive mood, which should manifest as greater amplitude differences between high, low, and neutral valence words compared with a negative mood induction. This reasoning also aligns with previous ERP studies, where mood effects are more consistently observed under positive mood inductions, likely because the broadened distribution of precision in positive states enhances the detectability of valence-related differences.

## Method

### Participants

Twenty-two right-handed native English speakers between 18 and 40 years (13 women, M = 29.8, SD = 7.1) participated in the study. All participants reported English as their dominant language in daily use; they had normal or corrected-to-normal vision, and reported no mood, psychiatric, or neurological disorders. Signs of depression, anxiety and excessive stress were checked for by means of Depression Anxiety Stress Scales (DASS-21) (Lovibond & Lovibond, 1995). Participant scores fell in the category of normal to mild for Depression and normal to moderate for Anxiety and Stress. The Positive and Negative Affect Schedule (PANAS) (Watson et al., 1988) was used to determine participants’ prevailing mood over the week preceding the experiment. None of the participants reported scores for both positive and negative affect that might be associated with depression. Table 1 summarises participant data.

**Table 1:** Participant information, with mean and standard deviation

Measurement and score			Level of proficiency (self-reported, on a scale from 1 to 10)			Current exposure to the language (degree on a scale from 1 to 10)		
	M	SD		M	SD		M	SD
Handedness	89.7	19.9	Speaking	9.5	0.7	Interaction with friends	8.3	2.6
Depression	6.6	4.2	Reading	9.6	0.7	Interaction with family	7.3	3.6
Anxiety	5.5	5.1	Listening	9.7	0.6	Reading	8.8	1.6
Stress	13.5	7.8	Age of acquisition (years)			Auditory information	7.8	2.1
Positive affect	32.2	7.5	Speaking	1	1.7			
Negative affect	18.6	5.4	Reading	4.3	1.3			

Units of measurement and source: Handedness from −100 (strong left-handedness) to 100 (strong right-handedness) (adapted from Oldfield, 1971). Depression, Anxiety and Stress, over the past week, from DASS-21 (Lovibond & Lovibond, 1995), reference scores: depression: normal (0–9), mild (10–13); anxiety: normal (0–7), mild (8–9), moderate (10–14); stress: normal (0–14), mild (15–18), moderate (19–25). Positive and Negative affect, over the past week, based on PANAS (Watson et al., 1988), reference mean scores: positive affect: 33.3 (7.2), negative affect: 17.4 (6.2). Linguistic information from the LEAP-Q (Marian et al., 2007).

The participants were compensated for their time with a cinema voucher. The study was approved by the Swedish Ethical Review Authority (approval number 2023-00811-01), and the participants gave written informed consent prior to the experiment.

### Verbal Stimuli

A total of 360 words (120 positive, 120 negative and 120 neutral) were selected from the Affective Norms for English Words (ANEW; Bradley & Lang, 2017) to serve as verbal stimuli. The selected words differed significantly in valence. Arousal, concreteness, frequency, and word length were controlled for (see Table 2 for mean values). The fluctuation of arousal scores is determined by the natural distribution of words within the ANEW, where all valenced words are higher in arousal than neutral ones. The 360 words were collapsed into three pseudorandomised blocks of 120 trials, each containing 40 low-valence, 40 high-valence, and 40 neutral words. Each block was further collapsed into three equal balanced subsets.

**Table 2:** Mean values for valence and other controlled variables for verbal stimuli

Variable	Positive words		Neutral words		Negative words	
	M	SD	M	SD	M	SD
Valence	7.3	0.5	5	0.6	2.6	0.6
Arousal	5	0.5	4.8	0.8	5	0.6
Concreteness	3.6	1.1	3.7	1.1	3.5	1
Word frequency (zipf)	4.1	0.7	4	0.7	3.9	0.6
Word length (syllables)	1.9	0.7	1.9	0.7	1.9	0.7
Word length (letters)	6	1.6	6	1.5	6	1.4

Units of measurement and source: Valence and Arousal on a scale from 1 (low) to 9 (high) from ANEW, Bradley & Lang (2017); Concreteness on a scale from 1 (abstract) to 5 (concrete) from Brysbaert et al. (2014); zipf frequencies from SUBTLEX_UK, Van Heuven et al. (2014); Syllables in number of syllables; Letters in number of letters.

### Mood induction

Mood induction was performed via images. Two sets of 60 affectively evocative pictures each were selected from the International Affective Picture System (IAPS; Lang et al., 2008). The images were controlled for arousal and differed in valence ratings (positive mood inducing images, arousal: M = 5, SD = 0.9, valence: M = 7.5, SD = 0.4; negative mood inducing images: arousal: M = 5.4, SD = 0.7, valence: M = 2.6, SD = 0.4).

The effects of mood induction via images have been shown to be relatively stable for 2 min, diminishing quickly after that (Kuijsters et al., 2016). For that reason, the procedure was performed in three blocks of 20 images, projected consecutively in random order for 5 s each, the total time of presentation equalled 100 s per block. Manipulation check was performed via a pen-and-paper version of the Self-Assessment Manikins (SAM; Bradley & Lang, 1994). A mood reset was performed prior to the end of the experiment by means of exposing the participants to another block of IAPS images high in valence.

At the beginning of the experiment, the participants rated their mood for valence and arousal. Those served as the baseline, or control, mood ratings. Mood induction was conducted three times in either mood, each time immediately followed by word evaluations and a subsequent mood manipulation check identical to that used to establish the baseline.

### Procedure

The experiment was performed at the Humanities Lab at Lund University, Sweden, in a single session. Upon arrival, the participants were familiarised with the setting; the purpose of the study was explained to them in general terms as an emotional language study. During electrode application, the participants filled in a few questionnaires: a personal information and selected medical history form, a modified Edinburgh Handedness Test (Oldfield, 1971), a modified Language Experience and Proficiency Questionnaire (LEAP-Q; Marian et al., 2007), DASS-21 (Lovibond & Lovibond, 1995), and PANAS (Watson et al., 1988). After the capping, the participants were given instructions for the experiment and performed a trial test consisting of ten words not used in the actual study.

The experiment had a within-subject design; hence each participant was engaged in three measurement sessions. The baseline condition session came first, followed by a positive or negative mood induced session (counterbalanced order). Before the first session, the participants assessed their valence and arousal ratings by using SAM (Bradley & Lang, 1994) to serve as the baseline mood score. In a block design, image presentation (*n* = 20) was followed by an emotional judgment task (*n* = 40), and a manipulation check was performed. The order of trial blocks and trials within each block was randomised.

During the experiment, the participants were comfortably seated in a normally lit room in front of a stationary Windows computer with a 24” monitor. In an emotional judgment task, they evaluated the word on a 5-point Likert scale, from least to most pleasant. They responded by pressing keys on the keyboard with their right hand. The words were presented one at a time in lowercase letters against a grey background. We chose a task that involves an explicit judgment of emotional valence to ensure deep semantic processing of emotional content of the words, as earlier research has reported more pronounced effects in such tasks as opposed to ones where valence is irrelevant (González-Villar et al., 2014). To maximise the effect of valence, we used a 5-point scale, as opposed to more common, simpler classifications into positive, negative and neutral words (Delaney-Busch et al., 2016; González-Villar et al., 2014; Kissler & Bromberek-Dyzman, 2021). A 7-point rating scale was previously implemented by Schacht and Sommer (2009); however, they shifted to a 5-point scale in a follow-up experiment to minimise EEG artifacts.

**Fig. 1:**
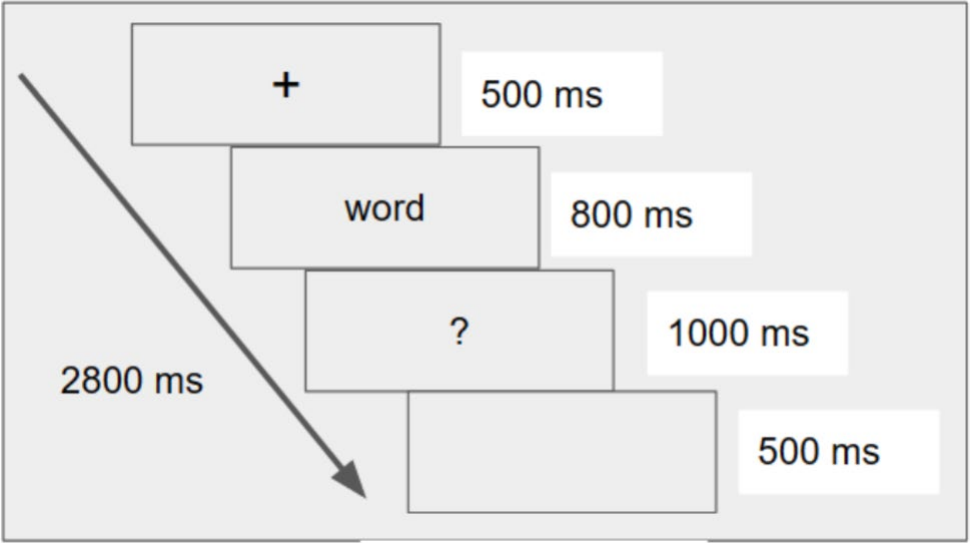
A single trial of a measurement session

The stimulus duration of 800 ms was followed by a question mark displayed for 1,000 ms, during which participants were instructed to evaluate the word. This was followed by an inter-trial interval (ITI) of 500 ms, during which a blank screen was displayed for 500 ms. This was followed by a fixation cross signalling the beginning of the next trial. The inter-stimulus interval (ISI) equalled 2 s. Figure 1 summarises the design of a single trial.

### EEG acquisition and processing

EEG signals were recorded from 62 scalp electrodes using Easycap and a Neuroscan SynAmps2 amplifier. The electrodes were placed following the extended International 10/20 System, with the ground at AFz. Two electrodes were placed at the canthi of the eyes to monitor horizontal eye movements and two above and below the left eye to monitor vertical movements. Two electrodes were placed on the mastoids. EEG was sampled at 1000 Hz/channel using Curry 7 by Neuroscan and referenced to the mastoid electrodes online. Impedances were kept below 5 kΩ for scalp electrodes, below 2 kΩ for the mastoids, and below 10kΩ for the eye electrodes. ERPs were time-locked to the word onset.

EEGLab (version 2022.1) (Delorme & Makeig, 2004) was used to analyse the data offline. During pre-processing, a bandpass filter was applied with a high-pass cut-off point at 0.05 Hz and a low-pass cutoff point at 30 Hz to remove high frequency noise. The scalp EEG channels were re-referenced to the average. Average reference was chosen based on literature (Kiefer et al., 2007; Kissler & Bromberek-Dyzman, 2021; Ogawa & Nittono, 2019), and due to the fact that the EPN is prominent near mastoid electrodes and can get subtracted if averaged mastoid reference is selected (Hajcak et al., 2012). Ocular artefacts were identified and attenuated using the Independent Component Analysis (ICA) through the function *runica* in EEGLab. Continuous data were segmented into epochs from a 200 ms baseline before stimulus onset to 800 ms poststimulus; baseline correction was performed. Epochs exceeding ±100 μV were removed. For two participants, 11% and 14% of epochs were rejected, and no more than 2% of the trials were rejected for the other participants. Overall, the number of rejected epochs across participants did not exceed 2%, with a total of 140 epochs rejected across a total of 7,560 trials.

### Analysis

#### Mood manipulation

To ensure that the manipulation was successful, we conducted nonparametric Friedman tests, because the data were not normally distributed. The normality of distribution was assessed by Shapiro-Wilks tests. Alpha was predefined at 0.05. Effect size was calculated through Kendall’s W test. Planned comparisons were made using pairwise Wilcoxon signed-rank tests separately for arousal and valence between the control mood and each of the mood inductions, as well as the participant average rating.

#### Behavioural data

We calculated response times (RT) to the words across conditions. To minimise movement artifacts while performing a relatively complex task, participants were instructed to make a delayed response at the question mark that followed the word. Responses that took longer than 1,500 ms after the onset of the question mark or 2,300 ms after the stimulus onset were not included in the analysis, which eliminated 22.4% of the trials. Mean response time value was calculated for each participant separately for each of the three moods, and the responses above and below two standard deviations of the participant’s mean RT were discarded. This eliminated another 3.6% of the original dataset, leaving a total of 5,864 trials, or an average of 267 trials per participant and 652 per condition.

To find a main effect of mood or valence, the data were analysed by using a linear mixed-effects model (Baayen et al., 2008). Mood and valence were included as categorical predictors with three levels: mood (control, positive, negative) × valence (neutral, high, low). The model included fixed effects for valence and mood, as well as their interaction, and it specified by-participant random intercepts and random slopes for mood to account for individual variability in the mood effect. Treatment coding was used and the baseline was set to control mood and neutral valence.

#### ERP

Based on previous literature on the effects of valence and mood on language processing, the following consecutive time-windows were selected: P1 (80–130 ms), N1 (130–190 ms), and EPN (200–300 ms) (Herbert et al., 2008; Herbert, 2022; Kissler et al., 2009; Kissler & Bromberek-Dyzman, 2021; Naranowicz et al., 2022b; Sass et al., 2010; Scott et al., 2009; , 2022b; Taylor et al., 2024; Zhang et al., 2017; T 2024). A component may peak at different times depending on the electrode site and experimental condition (Jia et al., 2022; Scott et al., 2009). That could be because visible peaks in the wave do not necessarily correspond to distinct ERP components (Kappenman & Luck, 2012) but are rather a weighted sum of local voltage changes caused by a chain of neurocognitive processes. Because neural processes may be prolonged in time, a partial or complete overlap might occur in the signature of ERP components, affecting the amplitude and shape of the peak. This is especially true for components with a broad distribution, such as N1, which is prone to be affected by the subsequent P2. Depending on scalp distribution, the shape and latency of the peak will differ.

**Fig. 2:**
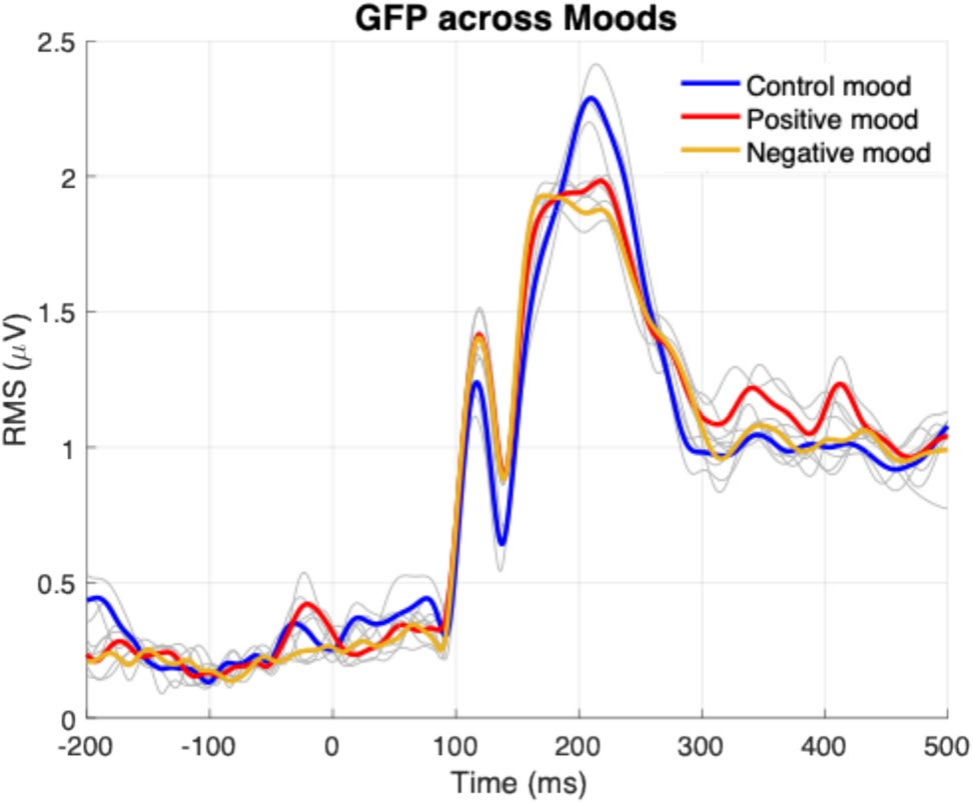
GFP of scalp electrodes in control (blue), positive (red), and negative (yellow) mood. Fine grey lines indicate GFP per individual condition; thick coloured lines show the average per each mood condition. GFP = global field power

To ensure that the preselected time windows are consistent with the otherwise data-driven approach, we performed a global field power (GFP) analysis (Lehmann & Skrandies, 1980) of averages across all participants, all epochs per condition, and across all electrodes except EOG. Reflecting global neural engagement, GFP analysis is widely used as an objective validation of component timing. The latency of ERP components can be regarded as the moment in time during which a large number of neurons are synchronized, manifesting as either a peak or a trough in electrical activity (Hamburger & Burgt, 1991). We found four major deflections in the GFP waveform: peaks at approximately 120, 170, and 215 ms, and a trough at 140 ms (Fig. 2). Their latencies are compatible with the P1, N1, and P2 components. This was consistent with previous literature, and well aligned with the preselected time windows. Peak and trough latencies per condition are given in supplementary materials.

Nonparametric cluster-based permutation tests (Oostenveld et al., 2011) were performed for the selected time windows and components to determine an unbiased scalp distribution. Instead of assuming a normal distribution of the collected data, permutation tests construct the empirical null distribution of the test statistic from the sample data. The Monte-Carlo method was used, 10,000 iterations were performed, and the Alpha level was set to 0.05. Permutation tests were conducted in MatLab (version 24.2.0.2863752 (R2024b) Update 5). Due to the explanatory nature of the study, we only did binary interactions. Comparisons on two levels would clearly indicate the direction of the interaction, whereas three-level comparisons would not.

If one or more clusters were found in the permutation test, we performed linear mixed-effects model analyses of the single trial data. Fixed effects included a main effect of mood and valence as well as their interaction. Mood and valence were included as categorical predictors with three levels: mood (control, positive, negative) × valence (neutral, high, low). The maximal model included by-subject and by-item random intercepts and random slopes for mood by participant to account for individual differences in how participants responded to mood conditions. Treatment coding was used and the baseline was set to control mood and neutral valence. If the maximal model did not converge, the random slope was removed (Barr et al., 2013). In five of six cases, the more complex model showed a singular fit, resulting in the choice of a simpler model with the slope for mood by participant dropped. We were interested in seeing whether there exists an interaction between any levels of the factors.

The analyses were performed using the *lme4* and *LmerTest* packages (Bates et al., 2015; Kuznetsova et al., 2017) in R (version 4.4.3 (2025-02-28)) via RStudio (version 2024.12.1+563) (R Studio Team, 2020). Post-hoc analyses performed via the *emmeans* package (version 1.8.5) (Lenth, 2023) are found in the supplementary data.

## Results

### Mood manipulation

**Fig. 3:**
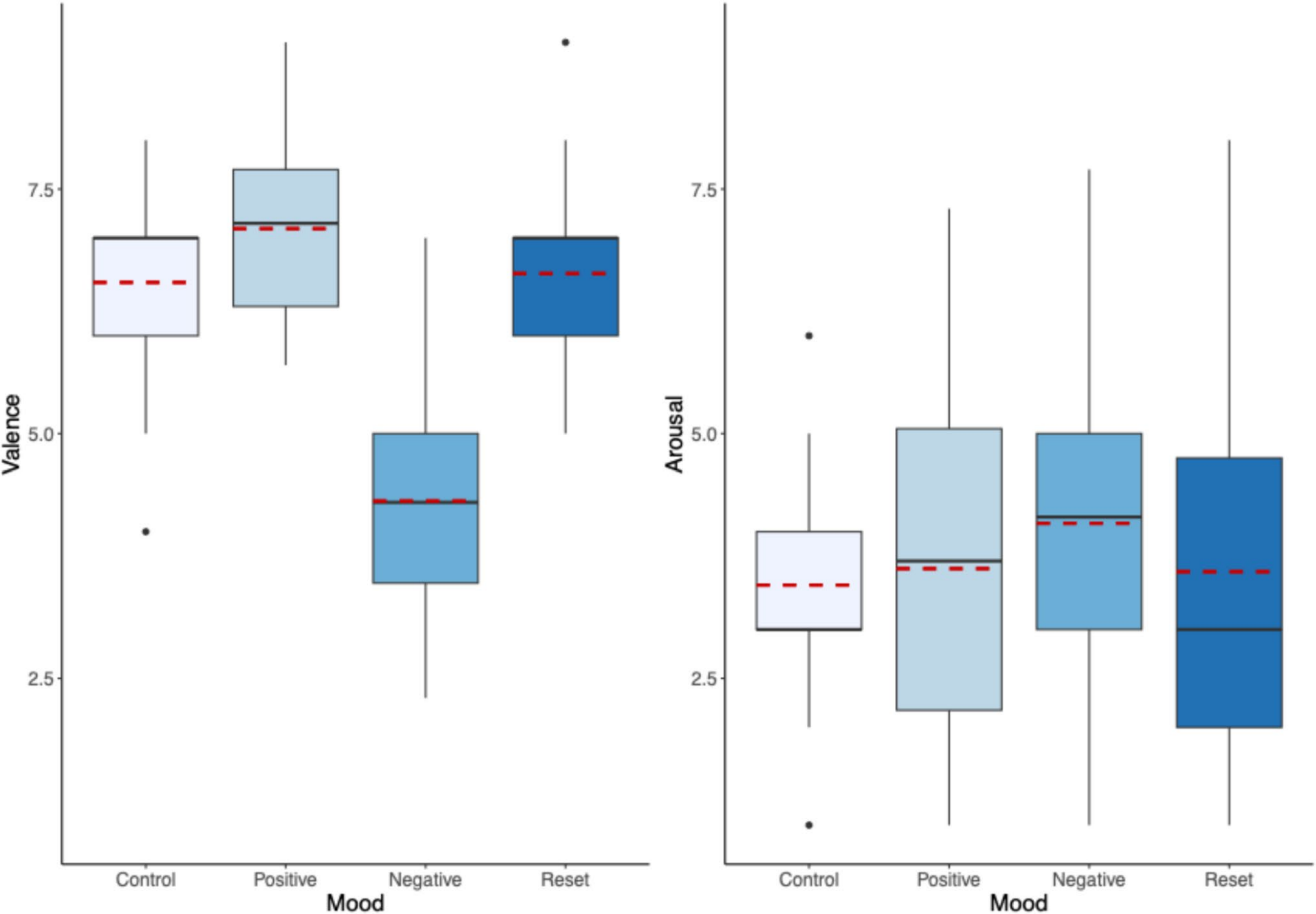
Valence and arousal scores across mood conditions. Valence and arousal on a scale from 1 to 9. The dashed red line represents the mean. The thick black line represents the median – the middle value in the dataset separating the higher end of the population sample from the lower one

The data from participants’ mood self-report after mood induction was not normally distributed (*p* > .05). For that reason, nonparametric Friedman tests with mood as within-subjects factor were conducted. The distributions of values across mood conditions is visualised in Fig. 3, and the means across conditions and the planned contrasts following Friedman test are listed in Table 3..

**Table 3:** Mean values and Friedman test results on mood manipulation

	Mean valence	Mean arousal	Valence	Arousal
Omnibus effect			χ^2^ = 37.4, *p* < .001, W = 0.57	χ^2^ = 2.41, *p* = .49, W = 0.04
Control	6.5	3.5		
Positive mood				
Induction 1	7.1	3.7	z = 28, *p* = .016	z = 26, *p* = .31
Induction 2	7.1	3.7	z = 59.5, *p* = .041	z = 41, *p* = .47
Induction 3	7.0	3.5	z = 25.5, *p* = .043	z = 40.5, *p* = .94
Average	7.1	3.6	z = 51, *p* = .015	z = 45, *p* = .41
Negative mood				
Induction 1	4.3	3.9	z = 190, *p* < .001	z = 46.5, *p* = .15
Induction 2	4.4	4.1	z = 210, *p* < .001	z = 19.5, *p* = .04
Induction 3	4.2	4.2	z = 190, *p* < .001	z = 35, *p* = .03
Average	4.3	4.1	z = 210, *p* < .001	z = 56, *p* = .07
Positive average vs. Negative average			z = 253, *p* < .001	z = 59, *p* = .15
Mood reset	6.6	3.6	z = 64.5, *p* = .87	z = 63, *p* = .81

Friedman tests found an omnibus effect of valence and no effect of arousal across levels of mood (control, positive, negative, reset). Positive mood was rated higher than in the control (*p* < .044) or negative condition (*p* < .001), although the difference between control and positive conditions in the self-reports was modest. Negative mood was successfully induced (*p* < .001) by all three blocks of emotional images. After the second and third inductions, arousal also differed significantly between the control and negative mood, introducing a potential confound.

### Response times

**Fig. 4:**
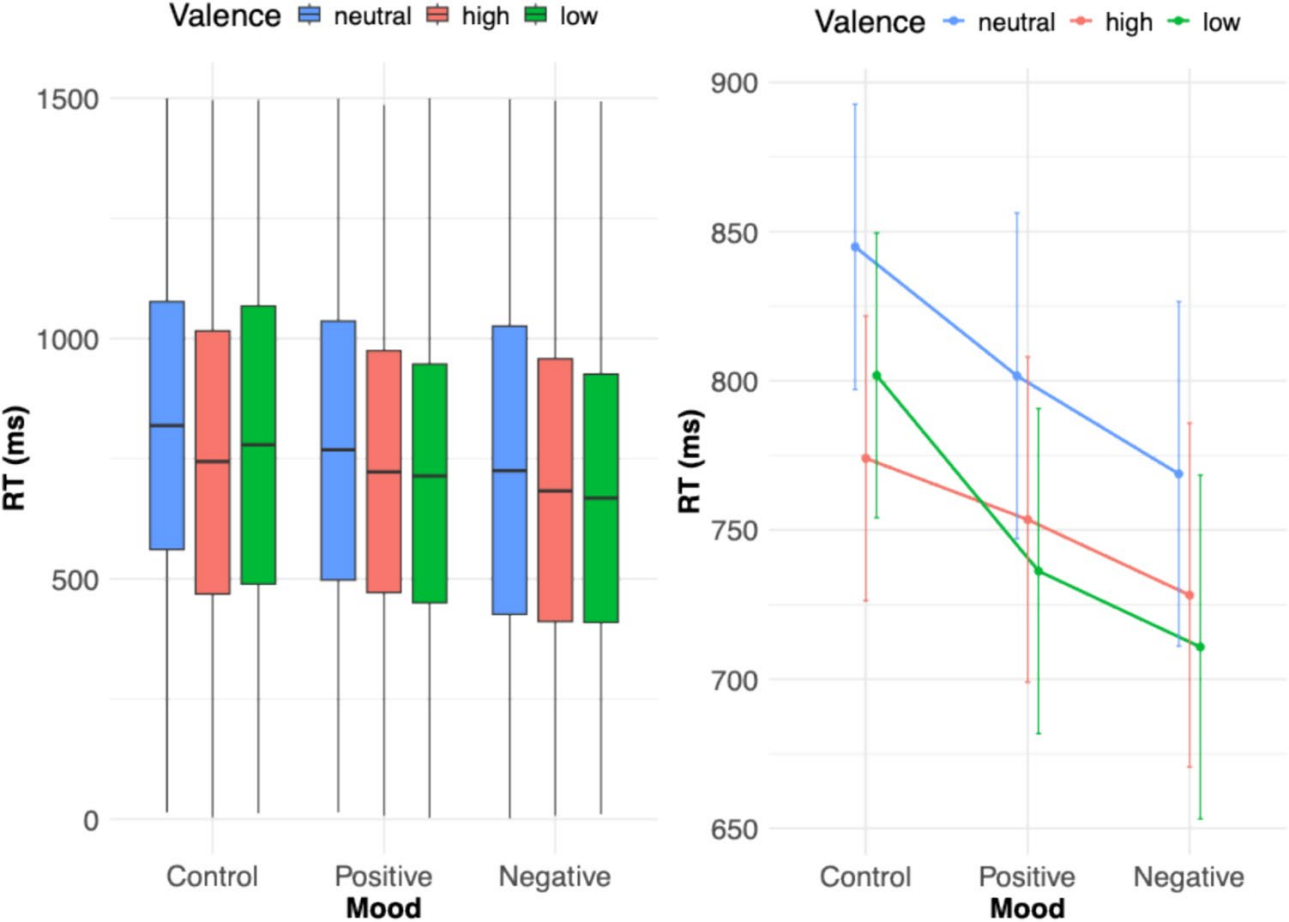
Response times across conditions. The box plot shows the distribution of RTs across moods: control (right), positive (centre) and negative (left). The interaction plot shows mean values to high-valence (red), low-valence (green) and neutral (blue) words in control, positive and negative moods. Error bars indicate standard error

A linear mixed-effects model was fitted with RT as the outcome, valence (neutral, high, low), mood (control, positive, negative) and their interaction as fixed effects, and by-participant random intercepts and random slopes for mood. The model revealed a significant effect of valence on RTs. Compared with neutral words, both high-valence (β = −70.89 ms, t(5802.65) = −4.64, *p* < .001) and low-valence words (β = −43.10 ms, t(5802.60) = −2.81, *p* = .005) elicited faster responses. The two valenced conditions did not differ significantly from each other. This was corroborated by paired comparisons of mean RTs across conditions. Across all three mood conditions, responses to valenced words were consistently faster than to neutral words (Fig. 4). In contrast, mood only showed a limited influence on RTs, with solely responses to low-valence words being significantly sped up by negative mood relative to control (β = −76.10 ms, t(29.89) = −2.15, *p* = .040). Thus, the analysis indicated a robust influence of valence on RT and a minimal effect of mood. Estimated means per condition are listed in Table 4. For model specification and contrasts, see supplementary material.

**Table 4:** Estimated mean response times per condition

Mood	Neutral valence	High valence	Low valence
Control mood	845 [722, 968]	774 [651, 897]	802 [679, 924]
Positive mood	802 [661, 942]	753 [613, 894]	736 [596, 876]
Negative mood	769 [620, 917]	728 [580, 876]	711 [562, 859]

95% confidence interval in brackets.

Valence judgment data are reported in the supplementary materials, Appendix 1.

### ERP

#### P1: 80–130 ms

No significant clusters were found in the permutation tests for interaction between mood (control, positive, negative) and valence (neutral, high, low) (*p* > .076).

#### N1: 130–190 ms

**Fig. 5:**
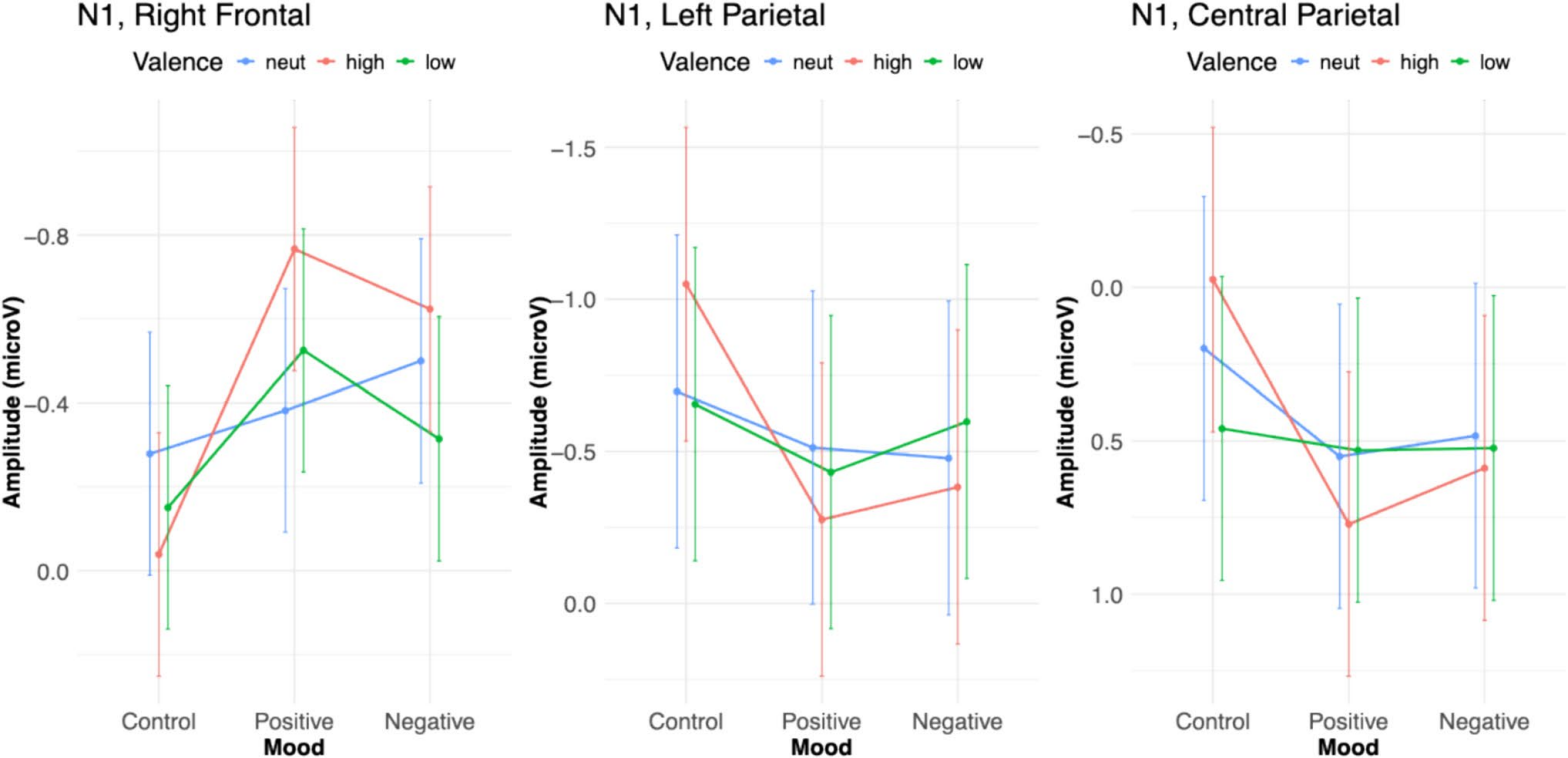
Mean amplitudes per condition over the N1 time-window (130−190 ms). Error bars for standard error

**Fig. 6:**
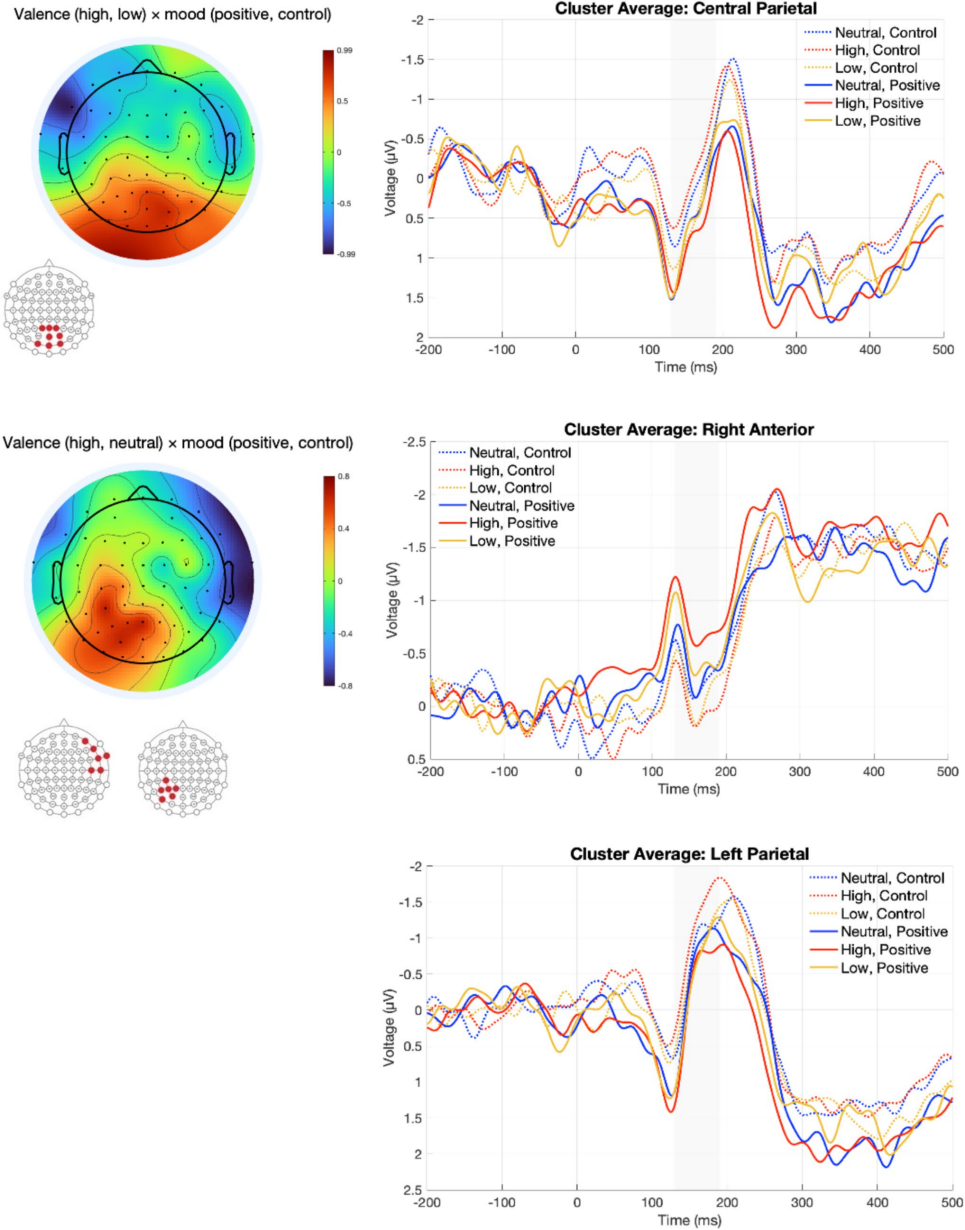
Scalp topographies (subtractions) and cluster averages in the time window of 130−190 ms. Valence: neutral (blue), high (red), low (yellow); mood: baseline (dotted lines), positive (solid lines). See supplementary materials for grand averages over individual electrodes

Over the time-window of 130–190 ms, high valence produced right frontal negative (*p* = .034) and left (*p* = .047) and central parietal positive (*p* = .007) effects in the positive mood, as seen in the permutation tests (interaction terms and electrodes are listed in Table 5). The linear mixed-effects model performed across three levels of mood and valence with an interaction and trial and participant as random factors confirmed the interaction over the clusters (see Table 5 for estimates). Significant mood differences emerged selectively for high-valence words. In the control condition, high valence elicited more pronounced responses than in the positive mood**,** with amplitudes more positive at the frontal site and more negative at the parietal sites (Figs. 5 and 6; ERP plots for all electrodes are given in supplementary material). No reliable mood effects were observed for neutral or low-valence words in any cluster. Model output as well as all contrasts and means are given in the supplementary material.

**Table 5:** Mood **×** valence interactions in the time window of 130–190 ms

**Cluster**	**Electrodes**	**Interaction as per permutation tests**	**Interaction, permutation tests**	**Interaction, LMM**
Right frontal, negative	F8, FT8, T8, AF8, C6, FT10	valence (high, neutral) **×** mood (positive, control)	*p* = .034	β = −0.62 ± 0.26, t(7553) = −2.37, *p* = .018
Left parietal, positive	CP3, P1, P3, P5, PO3, PO7	valence (high, neutral) **×** mood (positive, control)	*p* = .047	β = 0.59 ± 0.26, t(7537) = 2.24, *p* = .025
Central parietal, positive	P1/2, Pz, O1/2, Oz, POz, PO4	valence (high, low) **×** mood (positive, control)	*p* = .007	β = 0.73 ± 0.29, t(7557) = 2.54, *p* = .011

LMM = linear mixed-effects model.

#### P2/EPN: 200–300 ms

**Fig. 7:**
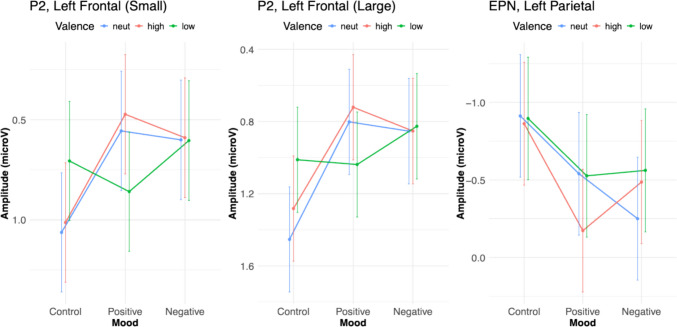
Mean amplitudes per condition over the P2/EPN time window (200–300 ms). Error bars for standard error

**Fig. 8:**
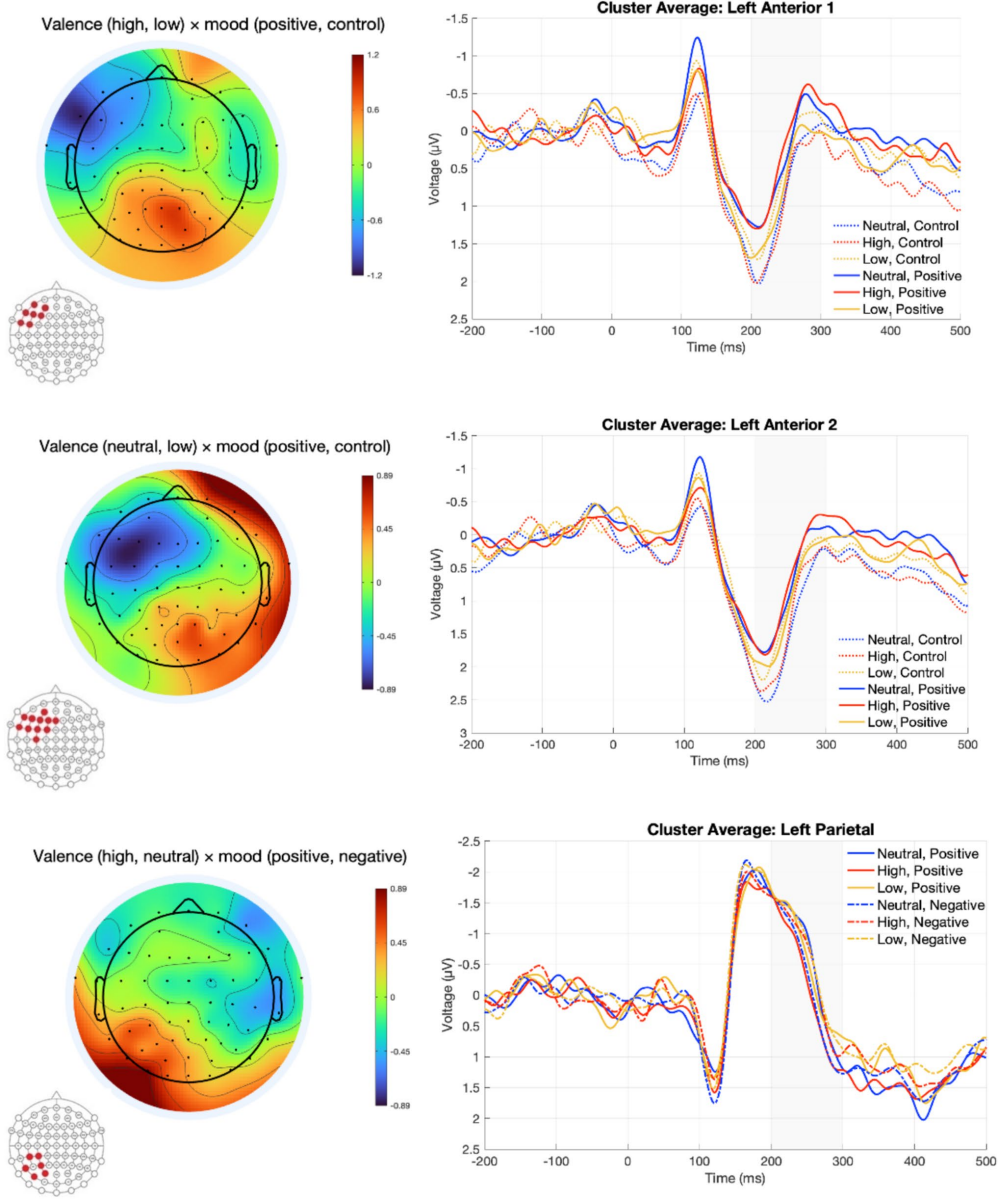
Scalp topographies (subtractions) and cluster averages in the time window of 200–300 ms. Valence: neutral (blue), high (red), low (yellow); mood: baseline (dotted lines), positive (solid lines), negative (dashed lines). See supplementary materials for grand averages for individual electrodes

Over the time window of 200−300 ms, high valence yielded left frontal negative (*p* = .027) and left parietal positive (*p* = .039) effects in the positive mood, as shown by the permutation test. Neutral words also elicited a left frontal negativity (*p* = .007) in the positive mood over a cluster that partially overlapped with the one where high-valence effects were found (the interaction terms and electrodes are listed in Table 6). The linear mixed-effects model confirmed the interaction over the clusters (see Table 6 for estimates). Mood selectively affected the amplitudes of high-valence and neutral words. In the positive mood, they produced a greater frontal negativity as opposed to control (Figs. 7 and 8; ERP plots for all electrodes, as well as contrasts and means are given in Supplementary material). The pattern was repeated in the negative mood. In addition, parietally, high-valence words produced a greater positivity in the positive mood as opposed to control, and neutral words elicited more positivity in the negative mood than in control.

**Table 6:** Mood **×** valence interactions in the time window of 200−300 ms

Cluster	Electrodes	Contrasts as per permutation tests	Interaction, permutation test	Interaction, LMM
Left frontal, negative	AF7, AF3, F7, F5, F3, FT7, FC5	Valence (high, low) **×** mood (positive, control)	*p* = .027	β = −0.69 ± 0.23, t(7565) = −3.02, *p* = .003
Left frontal, negative	AF3, F7, F5, F3, F1, Fz, FT7, FC5, FC3, FC1, C3	Valence (neutral, low) × mood (positive, control)	*p* = .007	β = −0.68 ± 0.22, t(7571) = −3.10, *p* = .002
Left parietal, positive	CP5, CP3, P7, P3, PO7, PO3	Valence (high, neutral) × mood (positive, negative)	*p* = .039	β = 0.60 ± 0.24, t(7596) = 2.51, *p* = .012

LMM = linear mixed-effects model.

## Discussion

The study investigated the time flow of the interaction between mood and valence during visual word processing in the brain. We tracked the neurophysiology of valence processing across moods in an emotional judgment task over three consecutive time windows. Participants rated individually presented words in terms of degree of pleasantness in two induced moods and an uninduced control mood.

The results indicate modulations of both prediction error and model updating in the presence of mood hyperpriors. The N1 component (130–190 ms after word presentation) was reduced for high-valence words in the positive mood, manifesting as a smaller left parietal negativity and right frontal positivity compared to baseline. We interpret this effect as a diminished prediction error for mood-congruent words in the presence of a strong hyperprior in the positive mood. The subsequent P2 component (200–300 ms) was selectively modulated in the positive mood, with high-valence and neutral words eliciting a reduced left frontal positivity compared to control. In parallel, the EPN (200–300 ms) showed a reduced posterior negativity for high-valence words in the positive mood and for neutral words in the negative mood, as compared to control. These effects might reflect model update: following a diminished prediction error observed at the N1 stage, the system adjusts its internal representation of word valence in light of the prevailing mood hyperprior. This means a reduced need for subsequent model update, which is contingent on the congruence or relevance of the stimulus with respect to mood state. Behavioural data corroborate the ERP findings. Facilitation for valenced as opposed to neutral words in induced moods could be a result as faster decision-making due to a reduced mismatch and limited need for update.

Mood induction was successful as seen in self-reported ratings. The strength of the induction in the positive mood was modest, but significant. The fact that RT and ERPs showed different patterns between mood-induced and control conditions also testifies to the success of the procedure.

From a predictive coding perspective, where attention is understood as increased precision in prediction (Friston, 2009), the pattern of reduced amplitudes in non-neutral moods reflects mood-dependent modulation of precision. Positive mood generates a prior that expects with high precision to encounter predictable input, which manifests itself in suppressed N1, P2, and EPN responses for congruent, high-valence words. By the P2 window, the precision weighting extends to neutral words, widening the range of inputs treated as predictable. Low-valence words continue to elicit larger amplitudes, pointing at a greater mismatch and subsequent error correction demands.

### ERP

We found congruence effects in the positive but not in the negative mood. The interaction between mood and valence mainly involved high-valence words and, to a certain extent, neutral words, at a later stage. The negative mood interacted with valence during later processing, to a smaller extent than the positive mood.

The general ERP pattern over posterior sites consisted of a first prominent positivity peaking at approximately 130 ms, followed by a negativity between 160–210 ms, and a subsequent sustained positivity reaching its maximum between 260 and 310 ms. Over anterior sites, as expected for components with broad generators and opposite field distributions (Hajcak et al., 2012; Luck & Kappenman, 2012), the same sequence appeared with reversed polarity: a negative peak around 130 ms, a subsequent positivity around 160 over the right anterior cluster and around 210–220 ms over the left hemisphere, and a later negativity peaking at around 270 ms, with an earlier onset over the right hemisphere. The observed pattern is consistent with reports of processing decontextualised words (Hauk et al., 2006; review in Pulvermüller et al., 2009). We believe this to be evidence of a single topographically and temporally coherent event in each time window.

This polarity inversion across the scalp is well-documented for the P1-N1 complex (Hauk et al., 2006) and is thought to indicate a coherent set of underlying neural processes rather than distinct components. Importantly, these anterior-posterior differences mean that visible peaks will not align perfectly across clusters, even though they reflect the same components (Luck & Kappenman, 2012). Global field power analyses corroborated this temporal organisation, showing consistent early peaks at 115–120 ms, troughs at 135–140 ms, and later maxima around 160–220 ms across conditions.

The earliest interaction between mood and valence was found in the N1 time window (130–190 ms). High-valence words decreased the N1, as seen in a right frontal negativity and left-central parietal positivity in both induced moods compared with control. The reduced negativity produced by high-valence words parietally occurred over the cluster of electrodes where N1 effects are commonly observed during visual processing of emotional stimuli (Ernst et al., 2013; Herbert, 2022; Simon et al., 2007; Zhang et al., 2014; 2017). High valence contrasts both low and neutral words in this time frame. High-valence effects in induced moods as opposed to control signal that words may already be discriminated based on their valence during early visual processing, in line with some previous studies (Herbert, 2022; Kissler & Herbert, 2013; Kissler et al., 2009; Scott et al., 2009). This corroborates the hypothesis that emotional features of words are accessed prior to or in parallel with lexical properties.

In the P2 time window, between 200 and 300 ms, neutral and high-valence words elicited a greater positivity in the control condition as opposed to both positive and negative moods over a left anterior region. Increased P2 amplitudes in response to positive words were previously found by Kanske & Kotz (2007) and explained as a manifestation of a general positivity bias (Kissler et al., 2006). A healthy population is believed to have a slight positivity bias during the perception of the environment, which manifests itself in a tendency to interpret situations that are neutral as mildly positive (Cacioppo et al., 1999). This positivity offset is accounted for by the evolutionary value of the motivation to explore, which is associated with positive affect, as opposed to avoidant tendencies when experiencing negative affect. Attention bias stands for increased attention allocation to positive stimuli, also for evolutionary reasons, since positive stimuli are more rewarding and hence trigger approach tendencies. That is to say that the positivity offset (Cacioppo et al., 1999) and a general attention bias to positive stimuli (Kress & Aue, 2017), taken together, could explain the greater amplitudes elicited by both neutral and positive words in the control mood.

Finally, mood did not affect the perception of emotional words in a way that was different from neutral ones in the P1 time window (80–130 ms). Most studies of emotional valence in language have also failed to detect differential processing during the traditional time window of P1 (Herbert et al., 2008; Herbert, 2022; Kissler et al., 2009; Pauligk et al., 2019; Schindler et al., 2018; Zhang et al., 2017). When preferential processing of emotional valence in words did influence the P1 in previous research, the component had a considerably later onset (Zhang et al., 2014), was only evoked by threatening stimuli among males but not females (Sass et al., 2010) or was mainly elicited by high but not low frequency words (Scott et al., 2009). Schindler et al. (2023), who found an early emotion effect in the conventional time window of P1 during visual word processing, call for caution in interpreting positive results due to an abundance of negative data in earlier research. Early visual attention effects may be a result of an interaction of emotion with other factors, such as word frequency (Palazova et al., 2011; Scott et al., 2009) or participant gender (Sass et al., 2010), and mood may not be one of those.

Taken together, the results highlight that the interaction between mood and valence is present only at specific levels rather than globally. High-valence words were prone to being affected by mood starting at 130 ms, resulting in a significant change in ERP amplitudes. Neutral words showed amplitude changes in the P2 window, taking on the same pattern as high-valence words. Low-valence words, however, were not affected by mood shifts, at least until 300 ms. The valence of mood played an effect in the interaction at the earlier processing stage, the difference essentially being between control and non-neutral moods during in a later time window. Certain similarities observed between positive and negative moods in our results might be due to the fact that negative mood inductions do not necessarily strengthen negative expectations but rather diminish positive affect, as has been highlighted in earlier work (Joseph et al., 2020), which leads to overlapping processing patterns in positive and negative mood conditions.

Interpreted from a predictive coding perspective, the results suggest that mood shapes both the generation of predictions and the updating of the model in response to error. The early N1 reduction for high-valence words in the positive mood can be interpreted as a diminished prediction error for mood-congruent input, reflecting reduced surprise in the presence of strong hyperpriors. Following this, the P2 appears to index selective model updating: input that aligns with the mood elicits reduced amplitudes, indicating a decreased need for revision of the model. By contrast, low-valence words maintain larger amplitudes, suggesting that they remain mismatched to the model and require error correction.

Literature on the auditory modality has associated the N1 and P2 suppression with successful prediction generation at different processing stages (Knolle et al., 2019; Schröger et al., 2015; review in Roll et al., 2023). Our findings suggest that reduced N1 and P2 amplitudes can also be viewed as indicators of a better-fitted model during visual processing. N1 indexes prediction error elicited by incongruent input, and an early reduction of surprise for mood-congruent words. P2 reflects subsequent incorporation of this input into the generative model—an index of model update. The later EPN effects reinforce this interpretation: in the positive mood, high-valence words were down-weighted due to their consistency with optimistic priors, while in the negative mood, neutral words were attenuated, consistent with a model that biases attention toward volatility and threat-consistent evidence. In sum, these results support the idea that mood selectively tunes precision allocation, amplifying mood-congruent signals and attenuating incongruent ones, and that the scope of what is treated as congruent or reliable depends on the affective state of the system.

An interpretation of the results within the framework of most previous studies on mood and emotion might be based on the hypothesis that positive and negative moods, respectively, promote top-down, schema-driven and bottom-up, input-reliant processing. Valence discrimination is seen as enhanced attention allocation to particular properties of the stimulus across processing stages: automatic attention focus during the N1 time window, exogenous attention to the stimulus features indicated by the anterior P2 (Carretié, 2014; Kanske et al., 2011), and facilitated or impeded disengagement from the stimulus reflected in the EPN slope. The results suggest that mood selectively modulates how attention is allocated to emotional and neutral words across processing stages. The early N1 reduction for high-valence words in both positive and negative moods indicates an automatic shift of attentional resources toward emotionally salient stimuli, which does not occur in the control state. By the P2 stage, this effect broadens to include neutral words, while low-valence words appear resistant to modulation, suggesting that their processing relies on a more stable allocation of attentional resources that is less affected by transient mood states. The later EPN results show that mood continues to shape attentional engagement, with positive mood reducing sustained processing of high-valence words and negative mood reducing sustained processing of neutral words. Together, these findings support the idea that mood does not exert a uniform influence but rather tunes attentional allocation dynamically, depending on the valence of the stimuli and the stage of processing.

Both predictive and socio-cognitive frameworks capture important aspects of the results, such as the selective nature of mood effects on valence processing, but differ in the focus and depth of interpretation. The traditional account interprets the findings as mood-dependent changes in processing styles, which manifest themselves in differences in attention allocation. Attention is understood as selective distribution of limited resources, and it is the role of mood to determine whether these resources are allotted to the general picture, or whether details are attended to. The predictive coding account, by contrast, explains the same ERP patterns as the result of mood acting as a hyperprior that shapes prediction error and precision-weighting. Predictive frameworks regard attention as increased precision in prediction, and mood determines which signals are treated as reliable. Rather than describing separate processing styles, predictive coding frames mood as tuning the expected reliability of input and thereby modulating both error detection (N1) and model update (P2, EPN). In sum, while the traditional view highlights broad cognitive styles describing processing under different moods, the predictive account offers an explanation of how mood alters the neural balance between prediction and sensory input.

### Behavioural results

High- and low-valence words, without showing a difference in RTs from each other, were characterised by faster responses than neutral ones across all three mood conditions. Mood only showed a limited effect; faster responses were observed for low-valence words in the negative mood. While these results confirm preferential processing of emotional words after mood induction, consistent with earlier research (González-Villar et al., 2014; Kissler & Bromberek-Dyzman, 2021; Schacht & Sommer, 2009), they only partially replicate reports of generally faster responses in induced moods compared with control (Sereno et al., 2015). We believe this may be due to the nature of the task used to collect behavioural measures.

Out task differs from typical semantic decision paradigms often used in emotional word research, which usually involve categorisations of words into pleasant, unpleasant and neutral (Delaney-Busch et al., 2016; González-Villar et al., 2014; Kissler & Bromberek-Dyzman, 2021). By asking participants to rate words on a continuous valence scale rather than assigning them to discrete categories, the task placed greater cognitive demands on evaluative processing. This more complex judgment procedure, combined with the instruction to produce delayed responses to minimise ERP artifacts, means that response times may likely reflect post-perceptual decision-making and memory maintenance. Consequently, the sensitivity of RTs as a direct index of early mood effects is reduced. At the same time, this task represents a novel contribution relative to prior binary decision paradigms, highlighting the neurocognitive processes involved in evaluating the degree of word valence and illustrating how mood modulates processing under more nuanced evaluative conditions.

Unlike the ERP findings, the behavioural data data did not clearly reflect interactions between mood and valence. While ERP amplitudes were modulated by specific mood-valence combinations during early processing, RTs instead showed a more general facilitation for high and low valence, and the effect of mood appeared to be additive to a generally faster processing of emotional words. This suggests that behavioural measures capture overall processing advantages for emotional words, but might be less sensitive to the fine-grained temporal dynamics and interaction effects revealed by ERP responses.

### Mood manipulation

Analysis of self-reported mood ratings across conditions indicate that the negative mood induction was considerably more successful than the positive one: although both were statistically reliable, the positive mood induction was very modest in magnitude. This is not a novel situation; the difference in magnitude between the two moods when induced has been reported extensively (Chwilla et al., 2011; Kiefer et al., 2007; Ogawa & Nittono, 2019; Van Berkum et al., 2013). The difficulty in inducing positive mood might be related to the fact that a healthy population is characterised by a mild positive affect (Cacioppo et al., 1999; Kress & Aue, 2017), so positive mood-inducing stimuli have to be particularly potent to overcome the positivity offset. In studies that contrasted positive and negative mood conditions, positive mood inductions often maintained the initially high valence (Sereno et al., 2015; Van Berkum et al., 2013) or resulted in a very modest albeit significant increase from the pre-induction baseline (Lai et al., 2022). Finally, both RTs and ERPs showed significant differences between the control and positive condition, which indicates that the positive mood induction was indeed successful. However, future research might consider the use of strong inducing stimuli for positive mood and an addition of alternative measures of testing for a successful induction other than participant self-report.

The positivity offset also affects how negative mood is operationalised in a healthy population. A score of 4.3 on a 9-point scale represents a highly significant decrease from the baseline and can be considered a successful negative mood induction even though it is in the middle of the scale. Similar negative scores have been previously reported (Cheng et al., 2017; Rowe et al., 2007; Sereno et al., 2015; Van Berkum et al., 2013), which underlines that it is the magnitude of the reduction rather than the absolute values that matters. A lower negative score might be achieved through changes to the mood-induction procedure, for example, using negative stimuli with a greater negativity from other pre-normed datasets, or by explicitly instructing participants to engage with the stimuli, based on the principles of emotion regulation (Gross, 2002).

After two blocks of negative mood induction, the arousal difference with the control was great enough for arousal, in principle, to be considered a potential confound to valence. However, this risk was minimized by both word lists and mood-inducing images being matched in arousal, so that the conditions differed only in valence. The arousal differences in self-reports were smaller than those caused by changes in perceived valence of one’s emotional state and not systematic across inductions, suggesting that the observed effects are primarily driven by valence rather than arousal.

## Conclusion

In this exploratory study, we investigated the interaction of mood and valence using electrophysiological measures. The results point at facilitating effects of positive moods in visual word processing, in a mood-congruent way. Interpreted from the predictive coding perspective, the data show that positive mood generates an internal model that is different from that in a neutral emotional state and is characterized by higher expectations for positive sensory input. The N1 amplitudes manifesting as left parietal negativity and right frontal positivity were lower in response to high-valence words in the positive mood compared to control, pointing at a reduced prediction error to words congruent with the hyperprior. P2 amplitudes seen as a left frontal positivity were reduced for high-valence and neutral words in the positive mood as opposed to control, which is indicative of reduced demands during model update. These findings support the theoretical framework that regards mood as a hyperprior regulating the generative model and precision-weighting. The study adds to a growing body of research on the interrelation of affect and cognition and contributes to a better understanding of the effects of mood in processing the emotional content of words in particular, and emotional input in general, and opens new perspectives on looking at visual processing through the lens of predictive coding.

## Supplementary Information

Below is the link to the electronic supplementary material.

Supplementary file1 (DOCX 4245 KB)

## Data Availability

The data and materials for this experiment are available at https://osf.io/j2enh/. This experiment was not preregistered.

## Code availability

R scripts are available at https://osf.io/j2enh/.

## Declarations

### Open Access (not listed in declarations in the journal submission guidelines)

Word and picture lists, scripts and datasets for this experiment are available at https://osf.io/j2enh/. EEG data are available upon request. This experiment was not preregistered.
