# Supplementary material for: Mood modulations of emotional word processing: A predictive perspective view of EEG data
Source: Cogn Affect Behav Neurosci. 2026 Mar 25;26(3):1101–22. doi: 10.3758/s13415-025-01394-x (PMC13260159; doi:10.3758/s13415-025-01394-x)
Supplement: Supplementary file 1 — Supplementary file1 (DOCX 4245 KB) [file 13415_2025_1394_MOESM1_ESM.docx]

**Supplementary material**

Appendix 1. Analysis of valence evaluations.

Appendix 2. Grand averages per condition across all electrodes of an interaction cluster.

Appendix 3. Global field power analysis.

Appendix 4. Linear mixed-effects models per cluster.

Appendix 5. Estimated marginal means and contrasts per cluster.

Appendix 6. Linear mixed-effects models on response times data.

Appendix 7. IAPS images used for mood induction.

**Appendix 1. Analysis of valence evaluations**

**Method**

Participants’ valence judgments were analysed using the same linear mixed-effects model as for the analysis of RT data (see Methods section). To clarify the pattern within each valence category, pairwise comparisons of estimated marginal means were conducted. To test for a presence of a correlation between word evaluations and self-reported mood, a correlation analysis was conducted using a non-parametric Spearman test.

**Both induced moods lowered the perceived valence of words compared with the control condition** (positive mood: β = -0.37, t(67.99) = -3.00, p = .004; negative mood: β = -0.54, t(56.23) = -4.12, p < .001)**.** The model also yielded a significant interaction between predefined valence and negative mood for the low valence category (β = 0.46, t(5878.17) = 3.50, p < .001), indicating a modest change in how low valence ratings shifted relative to the neutral reference level. However, simple-effects comparisons showed that low valence words themselves did not differ significantly across mood conditions (p > .55). In contrast, neutral words were clearly affected: both positive and negative mood produced lower ratings than control (positive mood: t(64.3) = 2.96, p = .012; negative mood: t(57.0) = 4.05, p < .001). For high valence words, only the contrast between control and sad mood approached significance (p = .058). Model output can be found in Table 1. Table 2 lists estimated marginal means of perceived valence per condition. Overall, mood reliably influenced neutral words, showed only weak evidence of an effect on high valence words, and did not meaningfully alter evaluations of low valence words (Figure 1, left).

To test for a presence of a correlation between word evaluations and self-reported mood, a correlation analysis was conducted using a non-parametric Spearman test. A moderate positive correlation was found between reported mood ratings and word evaluations (r(59) = 0.36, p = 0.004). A higher self-reported valence score correlated with a higher valence attributed to a word (Figure 1, right).

| **Table 1. Linear mixed-effects model** | |  |  | |  | |  | |
| --- | --- | --- | --- | --- | --- | --- | --- | --- |
| **Fixed effects** |  |  |  | |  | |  | |
|  | **Estimate** | **SE** | **df** | | **t-value** | | **p** | |
| Intercept: Valence(Neut), Mood(Control) | 5.20 | 0.13 | 34.18 | | 41.237 | | < 2e-16 * | |
| Valence (High) | 2.21 | 0.09 | 5879.57 | | 23.818 | | < 2e-16 * | |
| Valence (Low) | -2.38 | 0.09 | 5878.76 | | -25.515 | | < 2e-16 * | |
| Mood (Pos) | -0.37 | 0.12 | 67.99 | | -3.001 | | 0.004* | |
| Mood (Neg) | -0.54 | 0.13 | 56.23 | | -4.115 | | 0.0001* | |
| Valence(High):Mood(Pos) | 0.21 | 0.13 | 5875.91 | | 1.576 | | 0.115 | |
| Valence(Low):Mood(Pos) | 0.24 | 0.13 | 5875.28 | | 1.821 | | 0.069 | |
| Valence(High):Mood(Neg) | 0.23 | 0.13 | 5878.50 | | 1.768 | | 0.077 | |
| Valence(Low):Mood(Neg) | 0.46 | 0.13 | 5878.17 | | 3.502 | | 0.0005* | |
|  |  |  |  | |  | |  | |
| **Random effects** |  |  |  | |  | |  | |
| **Groups** | **Name** | **Variance** | **Std.Dev.** | |  | |  | |
| participant | Mood (Control) | 0.249 | 0.499 | |  | |  | |
|  | Mood (Pos) | 0.128 | 0.357 | |  | |  | |
|  | Mood (Neg) | 0.170 | 0.412 | |  | |  | |
| Residual |  | 2.800 | 1.673 | |  | |  | |
|  |  |  |  | |  | |  | |
| Formula:  perceived_valence ~ valence * mood + (1 + mood \| participant) | | | |  | |  | |  |
| Number of observations: 5913, groups: participant, 22 | | | |  | |  | |  |

**Table 2. Estimated marginal means and contrasts**

| **Valence** | **Mood** | **EM Mean** | **SE** | **df** | **95% CI Lower** | **95% CI Upper** |
| --- | --- | --- | --- | --- | --- | --- |
| Neutral | Control | 5.20 | 0.129 | 35.2 | 4.88 | 5.53 |
| Neutral | Positive | 4.84 | 0.125 | 35.9 | 4.52 | 5.15 |
| Neutral | Negative | 4.67 | 0.112 | 42.0 | 4.39 | 4.95 |
| High | Control | 7.41 | 0.127 | 33.7 | 7.09 | 7.73 |
| High | Positive | 7.25 | 0.124 | 34.3 | 6.94 | 7.56 |
| High | Negative | 7.11 | 0.110 | 39.1 | 6.83 | 7.38 |
| Low | Control | 2.83 | 0.127 | 34.0 | 2.51 | 3.15 |
| Low | Positive | 2.70 | 0.124 | 34.5 | 2.39 | 3.01 |
| Low | Negative | 2.75 | 0.110 | 39.0 | 2.48 | 3.03 |

********

***Figure 1.*** *Left: Perceived valence ratings in control (blue), happy (red) and sad (green) mood. Error bars indicate Standard Error. Right: correlation between self-reported mood and valence judgment.*

**Appendix 3. Grand averages per condition across all electrodes of an interaction cluster**

******

**Figure 2. N1 left frontal (F8, FT8, T8, AF8, C6, FT10)**

*Grand averages of all electrodes in the cluster. Positive-Control mood conditions are plotted. Mood: positive (solid lines), control (dotted lines). Valence: neutral (blue), high (red), low (orange)*

** ****

**Figure 3. N1 left parietal cluster (CP3, P1, P3, P5, PO3, PO7)**

*Grand averages of all electrodes in the cluster. Positive-Control mood conditions are plotted. Mood: positive (solid lines), control (dotted lines). Valence: neutral (blue), high (red), low (orange)*

******

**Figure 4. N1 central parietal cluster (P1/2, Pz, O1/2, Oz, POz, PO4).**

*Grand averages of all electrodes in the cluster. Positive-Control mood conditions are plotted. Mood: positive (solid lines), control (dotted lines). Valence: neutral (blue), high (red), low (orange)*

********

**Figure 5. EPN left parietal cluster (CP5, CP3, P7, P3, PO7, PO3).**

*Grand averages of all electrodes in the cluster. Positive-Control mood conditions are plotted. Mood: positive (solid lines), control (dotted lines). Valence: neutral (blue), high (red), low (orange)*

********

**Figure 6. P2 left frontal clusters (Cluster 1: AF7, AF3, F7, F5, F3, FT7, FC5. Cluster 2: AF3, F7, F5, F3, F1, Fz, FT7, FC5, FC3, FC1, C3)**

*Grand averages of all electrodes in the cluster. Positive-Control mood conditions are plotted. Mood: positive (solid lines), control (dotted lines). Valence: neutral (blue), high (red), low (orange)*

********

**Figure 7. P2 left frontal clusters (Cluster 1: AF7, AF3, F7, F5, F3, FT7, FC5. Cluster 2: AF3, F7, F5, F3, F1, Fz, FT7, FC5, FC3, FC1, C3).**

*Grand averages of all electrodes in the cluster. Negative-Control mood conditions are plotted. Mood: negative (solid lines), control (dotted lines). Valence: neutral (blue), high (red), low (orange)*

**Appendix 3. Global field power analysis**

***Table 3.*** *Peaks and troughs in GFP analysis: voltage in microVolts (latency in ms)*

| **Condition** | **Peak 1** | **Trough 1** | **Peak 2** | **Peak 3** |
| --- | --- | --- | --- | --- |
| Neutral Valence Control Mood | 1.115 (115) | 0.653 (137) | 1.777 (172) | 2.414 (215) |
| High Valence Control Mood | 1.191 (116) | 0.543 (136) | 1.892 (176) | 2.276 (206) |
| Low Valence Control Mood | 1.429 (119) | 0.710 (139) | 1.620 (173) | 2.200 (209) |
|  |  |  |  |  |
| Neutral Valence Positive Mood | 1.579 (119) | 0.882 (141) | 1.926 (178) | 1.986 (216) |
| High Valence Positive Mood | 1.345 (119) | 0.922 (139) | 1.851 (167) | 1.964 (208) |
| Low Valence Positive Mood | 1.453 (117) | 0.873 (139) | 1.989 (188) | 1.990 (222) |
|  |  |  |  |  |
| Neutral Valence Negative Mood | 1.521 (118) | 0.899 (141) | 1.976 (172) | 1.883 (219) |
| High Valence Negative Mood | 1.398 (120) | 0.859 (138) | 1.931 (170) | 1.835 (218) |
| Low Valence Negative Mood | 1.371 (116) | 0.857 (138) | 1.898 (165) | 1.908 (220) |

******

******

**Figure 8.** *GFP across moods. The fine coloured lines indicate valence conditions, the thick black line shows the average.*

**Appendix 4. Linear mixed models per cluster**

Relevant interactions and effects are underlined.

| **Table 4. N1, left parietal cluster** | |  |  |  |  |
| --- | --- | --- | --- | --- | --- |
| **Fixed effects** |  |  |  |  |  |
|  | **Estimate** | **SE** | **df** | **t-value** | **p** |
| Intercept: Valence(Neut), Mood(Control) | -0.696 | 0.503 | 25.088 | -1.384 | 0.1787 |
| Valence (High) | -0.352 | 0.191 | 2056.800 | -1.846 | 0.0651 |
| Valence (Low) | 0.041 | 0.190 | 1995.829 | 0.218 | 0.8278 |
| Mood (Pos) | 0.184 | 0.185 | 7552.988 | 0.990 | 0.3220 |
| Mood (Neg) | 0.218 | 0.188 | 7318.115 | 1.162 | 0.2451 |
| Valence(High):Mood(Pos) | 0.590 | 0.263 | 7537.045 | 2.239 | 0.0252* |
| Valence(Low):Mood(Pos) | 0.039 | 0.263 | 7553.400 | 0.151 | 0.8803 |
| Valence(High):Mood(Neg) | 0.448 | 0.267 | 7381.647 | 1.677 | 0.0936 |
| Valence(Low):Mood(Neg) | -0.161 | 0.266 | 7326.412 | -0.606 | 0.5444 |
|  |  |  |  |  |  |
| **Random effects** |  |  |  |  |  |
| **Groups** | **Name** | **Variance** | **Std.Dev.** |  |  |
| item | (Intercept) | 0.112 | 0.335 |  |  |
| participant | (Intercept) | 5.185 | 2.277 |  |  |
| Residual |  | 14.888 | 3.858 |  |  |
|  |  |  |  |  |  |
| Model: amplitude ~ valence * mood + (1 \| participant) + (1 \| item) | | | |  |  |
| Number of observations: 7659, groups: items, 360, participant, 22 | | | |  |  |

| **Table 5. N1: right frontal cluster** | | | |  |  |
| --- | --- | --- | --- | --- | --- |
| **Fixed effects** |  |  |  |  |  |
|  | **Estimate** | **SE** | **df** | **t-value** | **p** |
| Intercept: Valence(Neut), Mood(Control) | -0.278 | 0.284 | 33.776 | -0.981 | 0.3334 |
| Valence (High) | 0.240 | 0.188 | 2202.764 | 1.273 | 0.2031 |
| Valence (Low) | 0.128 | 0.187 | 2135.652 | 0.682 | 0.4953 |
| Mood (Pos) | -0.103 | 0.186 | 7569.256 | -0.554 | 0.5795 |
| Mood (Neg) | -0.221 | 0.188 | 7201.333 | -1.175 | 0.2401 |
| Valence(High):Mood(Pos) | -0.624 | 0.264 | 7553.314 | -2.365 | 0.0181* |
| Valence(Low):Mood(Pos) | -0.271 | 0.263 | 7569.458 | -1.029 | 0.3037 |
| Valence(High):Mood(Neg) | -0.363 | 0.266 | 7279.309 | -1.362 | 0.1734 |
| Valence(Low):Mood(Neg) | 0.057 | 0.266 | 7210.513 | 0.215 | 0.8299 |
|  |  |  |  |  |  |
| **Random effects** |  |  |  |  |  |
| **Groups** | **Name** | **Variance** | **Std.Dev.** |  |  |
| item | (Intercept) | 0.041 | 0.204 |  |  |
| participant | (Intercept) | 1.389 | 1.179 |  |  |
| Residual |  | 14.991 | 3.872 |  |  |
|  |  |  |  |  |  |
| Model: amplitude ~ valence * mood + (1 \| participant) + (1 \| item) | | | |  |  |
| Number of observations: 7659, groups: items, 360, participant, 22 | | | |  |  |

| **Table 6. N1: central parietal cluster** | | |  |  |  |
| --- | --- | --- | --- | --- | --- |
| **Fixed effects** |  |  |  |  |  |
|  | **Estimate** | **SE** | **df** | **t-value** | **p** |
| Intercept: Valence(Neut), Mood(Control) | 0.198 | 0.484 | 25.867 | 0.410 | 0.6851 |
| Valence (High) | -0.224 | 0.204 | 2182.724 | -1.098 | 0.2721 |
| Valence (Low) | 0.260 | 0.203 | 2116.317 | 1.282 | 0.1999 |
| Mood (Pos) | 0.351 | 0.201 | 7567.469 | 1.747 | 0.0807 |
| Mood (Neg) | 0.284 | 0.203 | 7209.084 | 1.398 | 0.1622 |
| Valence(High):Mood(Pos) | 0.445 | 0.285 | 7551.507 | 1.559 | 0.1191 |
| Valence(Low):Mood(Pos) | -0.280 | 0.285 | 7567.775 | -0.985 | 0.3246 |
| Valence(High):Mood(Neg) | 0.329 | 0.288 | 7287.576 | 1.142 | 0.2537 |
| Valence(Low):Mood(Neg) | -0.220 | 0.288 | 7219.760 | -0.766 | 0.4438 |
|  |  |  |  |  |  |
| **Random effects** |  |  |  |  |  |
| **Groups** | **Name** | **Variance** | **Std.Dev.** |  |  |
| item | (Intercept) | 0.055 | 0.236 |  |  |
| participant | (Intercept) | 4.717 | 2.172 |  |  |
| Residual |  | 17.511 | 4.185 |  |  |
|  |  |  |  |  |  |
| Model: amplitude ~ valence * mood + (1 \| participant) + (1 \| item) | | | |  |  |
| Number of observations: 7659, groups: items, 360, participant, 22 | | | |  |  |

| **Table 7. P2: left frontal, small** | |  |  |  |  |
| --- | --- | --- | --- | --- | --- |
| **Fixed effects** |  |  |  |  |  |
|  | **Estimate** | **SE** | **df** | **t-value** | **p** |
| Intercept: Valence(Neut), Mood(Control) | 1.062 | 0.291 | 29.441 | 3.648 | 0.00102** |
| Valence (High) | -0.049 | 0.162 | 2263.832 | -0.304 | 0.76105 |
| Valence (Low) | -0.356 | 0.161 | 2193.719 | -2.203 | 0.02767* |
| Mood (Pos) | -0.506 | 0.161 | 7575.450 | -3.135 | 0.00173** |
| Mood (Neg) | -0.461 | 0.162 | 7130.240 | -2.835 | 0.00460** |
| Valence(High):Mood(Pos) | -0.033 | 0.229 | 7559.553 | -0.147 | 0.88299 |
| Valence(Low):Mood(Pos) | 0.659 | 0.228 | 7575.646 | 2.883 | 0.00394** |
| Valence(High):Mood(Neg) | 0.038 | 0.231 | 7218.138 | 0.167 | 0.86711 |
| Valence(Low):Mood(Neg) | 0.359 | 0.230 | 7141.908 | 1.560 | 0.11885 |
|  |  |  |  |  |  |
| **Random effects** |  |  |  |  |  |
| **Groups** | **Name** | **Variance** | **Std.Dev.** |  |  |
| item | (Intercept) | 0.007 | 0.083 |  |  |
| participant | (Intercept) | 1.581 | 1.257 |  |  |
| Residual |  | 11.282 | 3.358 |  |  |
|  |  |  |  |  |  |
| Model: amplitude ~ valence * mood + (1 \| participant) + (1 \| item) | | | |  |  |
| Number of observations: 7658, groups: items, 360, participant, 22 | | | |  |  |

| **Table 8. P2: left frontal, large** | |  |  |  |  |
| --- | --- | --- | --- | --- | --- |
| **Fixed effects** |  |  |  |  |  |
|  | **Estimate** | **SE** | **df** | **t-value** | **p** |
| Intercept: Valence(Neut), Mood(Control) | 1.453 | 0.285 | 29.112 | 5.096 | 1.92e-05*** |
| Valence (High) | -0.171 | 0.155 | 2220.391 | -1.099 | 0.27182 |
| Valence (Low) | -0.441 | 0.155 | 2152.243 | -2.842 | 0.00452 |
| Mood (Pos) | -0.651 | 0.154 | 7570.804 | -4.217 | 2.50e-05*** |
| Mood (Neg) | -0.600 | 0.155 | 7171.412 | -3.849 | 0.00012*** |
| Valence(High):Mood(Pos) | 0.090 | 0.219 | 7554.811 | 0.412 | 0.68070 |
| Valence(Low):Mood(Pos) | 0.677 | 0.218 | 7571.039 | 3.100 | 0.00194** |
| Valence(High):Mood(Neg) | 0.170 | 0.221 | 7254.103 | 0.771 | 0.44065 |
| Valence(Low):Mood(Neg) | 0.414 | 0.220 | 7182.207 | 1.878 | 0.06043 |
|  |  |  |  |  |  |
| **Random effects** |  |  |  |  |  |
| **Groups** | **Name** | **Variance** | **Std.Dev.** |  |  |
| item | (Intercept) | 0.019 | 0.140 |  |  |
| participant | (Intercept) | 1.526 | 1.235 |  |  |
| Residual |  | 10.312 | 3.211 |  |  |
|  |  |  |  |  |  |
| Model: amplitude ~ valence * mood + (1 \| participant) + (1 \| item) | | | |  |  |
| Number of observations: 7658, groups: items, 360, participant, 22 | | | |  |  |

| **Table 9. EPN: left parietal** | |  |  |  |  |
| --- | --- | --- | --- | --- | --- |
| **Fixed effects** |  |  |  |  |  |
|  | **Estimate** | **SE** | **df** | **t-value** | **p** |
| Intercept: Valence(Neut), Mood(Control) | -9.125e-01 | 3.868e-01 | 2.663e+01 | -2.359 | 0.0259* |
| Valence (High) | 4.986e-02 | 1.753e-01 | 1.873e+03 | 0.284 | 0.7761 |
| Valence (Low) | 1.616e-02 | 1.749e-01 | 1.819e+03 | 0.092 | 0.9264 |
| Mood (Pos) | 3.724e-01 | 1.665e-01 | 7.531e+03 | 2.237 | 0.0253* |
| Mood (Neg) | 6.621e-01 | 1.693e-01 | 7.438e+03 | 3.910 | 9.3e-05*** |
| Valence(High):Mood(Pos) | 3.170e-01 | 2.360e-01 | 7.515e+03 | 1.343 | 0.1792 |
| Valence(Low):Mood(Pos) | -2.561e-03 | 2.356e-01 | 7.531e+03 | -0.011 | 0.9913 |
| Valence(High):Mood(Neg) | -2.857e-01 | 2.401e-01 | 7.483e+03 | -1.190 | 0.2341 |
| Valence(Low):Mood(Neg) | -3.273e-01 | 2.397e-01 | 7.443e+03 | -1.365 | 0.1722 |
|  |  |  |  |  |  |
| **Random effects** |  |  |  |  |  |
| **Groups** | **Name** | **Variance** | **Std.Dev.** |  |  |
| item | (Intercept) | 0.174 | 0.417 |  |  |
| participant | (Intercept) | 2.958 | 1.720 |  |  |
| Residual |  | 11.906 | 3.450 |  |  |
|  |  |  |  |  |  |
| Model: amplitude ~ valence * mood + (1 \| participant) + (1 \| item) | | | |  |  |
| Number of observations: 7658, groups: items, 360, participant, 22 | | | |  |  |

**Appendix 5. Estimated marginal means and contrasts per cluster**

Relevant contrasts are underlined.

**Table 10. Estimated Marginal Means (EMMs) per cluster**

| **N1. Left parietal cluster** | |  |  |  |  |  |
| --- | --- | --- | --- | --- | --- | --- |
| **Valence** | **Mood** | **EM Mean** | **SE** | **df** | **95% CI Lower** | **95% CI Upper** |
| Neutral | Control | -0.697 | 0.515 | 26.3 | -2.01 | 0.620 |
| Neutral | Positive | -0.513 | 0.515 | 26.3 | -1.83 | 0.804 |
| Neutral | Negative | -0.478 | 0.516 | 26.5 | -1.80 | 0.840 |
| High | Control | -1.050 | 0.515 | 26.4 | -2.37 | 0.268 |
| High | Positive | -0.276 | 0.515 | 26.3 | -1.59 | 1.041 |
| High | Negative | -0.383 | 0.516 | 26.5 | -1.70 | 0.936 |
| Low | Control | -0.655 | 0.515 | 26.3 | -1.97 | 0.662 |
| Low | Positive | -0.432 | 0.515 | 26.3 | -1.75 | 0.885 |
| Low | Negative | -0.598 | 0.516 | 26.5 | -1.92 | 0.721 |
|  |  |  |  |  |  |  |
| **N1. Right frontal cluster** | |  |  |  |  |  |
| **Valence** | **Mood** | **EM Mean** | **SE** | **df** | **95% CI Lower** | **95% CI Upper** |
| Neutral | Control | -0.2788 | 0.290 | 35.4 | -1.007 | 0.449 |
| Neutral | Positive | -0.3820 | 0.290 | 35.6 | -1.111 | 0.347 |
| Neutral | Negative | -0.4999 | 0.291 | 36.2 | -1.231 | 0.231 |
| High | Control | -0.0388 | 0.290 | 35.8 | -0.768 | 0.691 |
| High | Positive | -0.7666 | 0.290 | 35.5 | -1.495 | -0.038 |
| High | Negative | -0.6233 | 0.292 | 36.4 | -1.355 | 0.109 |
| Low | Control | -0.1506 | 0.290 | 35.6 | -0.879 | 0.578 |
| Low | Positive | -0.5251 | 0.290 | 35.5 | -1.254 | 0.203 |
| Low | Negative | -0.3144 | 0.292 | 36.3 | -1.046 | 0.417 |
|  |  |  |  |  |  |  |
| **N1. Central parietal cluster** | | | | |  |  |
| **Valence** | **Mood** | **EM Mean** | **SE** | **df** | **95% CI Lower** | **95% CI Upper** |
| Neutral | Control | 0.1988 | 0.495 | 27.1 | -1.065 | 1.46 |
| Neutral | Positive | 0.5506 | 0.496 | 27.2 | -0.714 | 1.82 |
| Neutral | Negative | 0.4832 | 0.496 | 27.3 | -0.783 | 1.75 |
| High | Control | -0.0254 | 0.496 | 27.2 | -1.290 | 1.24 |
| High | Positive | 0.7715 | 0.496 | 27.1 | -0.493 | 2.04 |
| High | Negative | 0.5886 | 0.497 | 27.4 | -0.678 | 1.86 |
| Low | Control | 0.4597 | 0.496 | 27.2 | -0.805 | 1.72 |
| Low | Positive | 0.5307 | 0.496 | 27.1 | -0.734 | 1.80 |
| Low | Negative | 0.5235 | 0.497 | 27.4 | -0.743 | 1.79 |

| **P2. Left frontal cluster (small)** | | | | | | |
| --- | --- | --- | --- | --- | --- | --- |
| **Valence** | **Mood** | **EM Mean** | **SE** | **df** | **95% CI Lower** | **95% CI Upper** |
| Neutral | Control | 1.063 | 0.297 | 30.8 | 0.310 | 1.82 |
| Neutral | Positive | 0.556 | 0.298 | 31.0 | -0.197 | 1.31 |
| Neutral | Negative | 0.601 | 0.298 | 31.3 | -0.154 | 1.36 |
| High | Control | 1.013 | 0.298 | 31.1 | 0.259 | 1.77 |
| High | Positive | 0.473 | 0.298 | 31.0 | -0.280 | 1.23 |
| High | Negative | 0.590 | 0.299 | 31.5 | -0.165 | 1.35 |
| Low | Control | 0.706 | 0.298 | 31.0 | -0.047 | 1.46 |
| Low | Positive | 0.859 | 0.298 | 31.0 | 0.106 | 1.61 |
| Low | Negative | 0.604 | 0.299 | 31.4 | -0.151 | 1.36 |
|  |  |  |  |  |  |  |
| **P2. Left frontal cluster (large)** | | | | | | |
| **Valence** | **Mood** | **EM Mean** | **SE** | **df** | **95% CI Lower** | **95% CI Upper** |
| Neutral | Control | 1.454 | 0.291 | 30.5 | 0.7162 | 2.19 |
| Neutral | Positive | 0.802 | 0.292 | 30.7 | 0.0638 | 1.54 |
| Neutral | Negative | 0.854 | 0.292 | 31.0 | 0.1139 | 1.59 |
| High | Control | 1.283 | 0.292 | 30.7 | 0.5438 | 2.02 |
| High | Positive | 0.721 | 0.291 | 30.6 | -0.0171 | 1.46 |
| High | Negative | 0.853 | 0.293 | 31.1 | 0.1125 | 1.59 |
| Low | Control | 1.012 | 0.291 | 30.6 | 0.2739 | 1.75 |
| Low | Positive | 1.039 | 0.291 | 30.6 | 0.300 | 1.78 |
| Low | Negative | 0.827 | 0.292 | 31.1 | 0.086 | 1.57 |
|  |  |  |  |  |  |  |
| **EPN. Left parietal cluster** | | | | | | |
| **Valence** | **Mood** | **EM Mean** | **SE** | **df** | **95% CI Lower** | **95% CI Upper** |
| Neutral | Control | -0.913 | 0.395 | 27.9 | -1.92 | 0.094 |
| Neutral | Positive | -0.540 | 0.395 | 28.0 | -1.55 | 0.467 |
| Neutral | Negative | -0.250 | 0.396 | 28.2 | -1.26 | 0.758 |
| High | Control | -0.863 | 0.396 | 28.0 | -1.87 | 0.145 |
| High | Positive | -0.173 | 0.395 | 27.9 | -1.18 | 0.834 |
| High | Negative | -0.486 | 0.397 | 28.3 | -1.50 | 0.523 |
| Low | Control | -0.896 | 0.395 | 28.0 | -1.90 | 0.111 |
| Low | Positive | -0.527 | 0.395 | 27.9 | -1.53 | 0.480 |
| Low | Negative | -0.562 | 0.396 | 28.2 | -1.57 | 0.447 |

**Table 11. Paired contrasts per cluster**

| **N1. Left parietal cluster** | |  |  |  |  |  |
| --- | --- | --- | --- | --- | --- | --- |
| **Valence** | **Contrast** | **Estimate** | **SE** | **df** | **t-value** | **p** |
| Neutral | Control-Pos | -0.1841 | 0.186 | 7560 | -0.990 | 0.5833 |
| Neutral | Control-Neg | -0.2189 | 0.189 | 7332 | -1.161 | 0.4766 |
| Neutral | Pos-Neg | -0.0348 | 0.189 | 7548 | -0.184 | 0.9815 |
| High | Control-Pos | -0.7741 | 0.187 | 7539 | -4.141 | 0.0001 |
| High | Control-Neg | -0.6670 | 0.190 | 7423 | -3.502 | 0.0013 |
| High | Pos-Neg | 0.1071 | 0.190 | 7573 | 0.565 | 0.8387 |
| Low | Control-Pos | -0.2237 | 0.186 | 7561 | -1.200 | 0.4530 |
| Low | Control-Neg | -0.0573 | 0.190 | 7329 | -0.302 | 0.9510 |
| Low | Pos-Neg | 0.1664 | 0.189 | 7564 | 0.879 | 0.6535 |
|  |  |  |  |  |  |  |
| **N1. Right frontal cluster** | |  |  |  |  |  |
| **Valence** | **Contrast** | **Estimate** | **SE** | **df** | **t-value** | **p** |
| Neutral | Control-Pos | 0.103 | 0.186 | 7576 | 0.554 | 0.8446 |
| Neutral | Control-Neg | 0.221 | 0.188 | 7216 | 1.173 | 0.4691 |
| Neutral | Pos-Neg | 0.118 | 0.189 | 7493 | 0.623 | 0.8076 |
| High | Control-Pos | 0.728 | 0.187 | 7555 | 3.884 | 0.0003 |
| High | Control-Neg | 0.585 | 0.190 | 7332 | 3.071 | 0.0061 |
| High | Pos-Neg | -0.143 | 0.190 | 7528 | -0.756 | 0.7300 |
| Low | Control-Pos | 0.375 | 0.187 | 7577 | 2.005 | 0.1111 |
| Low | Control-Neg | 0.164 | 0.190 | 7215 | 0.864 | 0.6629 |
| Low | Pos-Neg | -0.211 | 0.189 | 7516 | -1.113 | 0.5061 |

| **N1. Central parietal cluster** | |  |  |  |  |  |
| --- | --- | --- | --- | --- | --- | --- |
| **Valence** | **Contrast** | **Estimate** | **SE** | **df** | **t-value** | **p** |
| Neutral | Control-Pos | -0.35182 | 0.202 | 7574 | -1.746 | 0.1883 |
| Neutral | Control-Neg | -0.28438 | 0.204 | 7226 | -1.396 | 0.3430 |
| Neutral | Pos-Neg | 0.06743 | 0.205 | 7497 | 0.330 | 0.9418 |
| High | Control-Pos | -0.79682 | 0.203 | 7554 | -3.934 | 0.0002 |
| High | Control-Neg | -0.61392 | 0.206 | 7339 | -2.983 | 0.0081 |
| High | Pos-Neg | 0.18290 | 0.205 | 7531 | 0.892 | 0.6451 |
| Low | Control-Pos | -0.07099 | 0.202 | 7576 | -0.352 | 0.9341 |
| Low | Control-Neg | -0.06376 | 0.205 | 7224 | -0.311 | 0.9481 |
| Low | Pos-Neg | 0.00723 | 0.205 | 7519 | 0.035 | 0.9993 |

| **P2. Left frontal cluster (small)** | |  |  |  |  |  |
| --- | --- | --- | --- | --- | --- | --- |
| **Valence** | **Contrast** | **Estimate** | **SE** | **df** | **t-value** | **p** |
| Neutral | Control-Pos | 0.5064 | 0.162 | 7583 | 3.133 | 0.0049 |
| Neutral | Control-Neg | 0.4617 | 0.163 | 7149 | 2.831 | 0.0129 |
| Neutral | Pos-Neg | -0.0446 | 0.164 | 7458 | -0.273 | 0.9599 |
| High | Control-Pos | 0.5401 | 0.163 | 7562 | 3.323 | 0.0026 |
| High | Control-Neg | 0.4231 | 0.165 | 7277 | 2.567 | 0.0277 |
| High | Pos-Neg | -0.1170 | 0.164 | 7497 | -0.713 | 0.7560 |
| Low | Control-Pos | -0.1529 | 0.162 | 7584 | -0.944 | 0.6125 |
| Low | Control-Neg | 0.1020 | 0.164 | 7148 | 0.622 | 0.8082 |
| Low | Pos-Neg | 0.2549 | 0.164 | 7484 | 1.555 | 0.2656 |

| **P2. Left frontal cluster (large)** | |  |  |  |  |  |
| --- | --- | --- | --- | --- | --- | --- |
| **Valence** | **Contrast** | **Estimate** | **SE** | **df** | **t-value** | **p** |
| Neutral | Control-Pos | 0.6516 | 0.155 | 7578 | 4.215 | 0.0001 |
| Neutral | Control-Neg | 0.6002 | 0.156 | 7190 | 3.844 | 0.0004 |
| Neutral | Pos-Neg | -0.0514 | 0.157 | 7479 | -0.328 | 0.9425 |
| High | Control-Pos | 0.5614 | 0.155 | 7557 | 3.612 | 0.0009 |
| High | Control-Neg | 0.4295 | 0.158 | 7310 | 2.723 | 0.0178 |
| High | Pos-Neg | -0.1319 | 0.157 | 7514 | -0.839 | 0.6787 |
| Low | Control-Pos | -0.0264 | 0.155 | 7579 | -0.170 | 0.9842 |
| Low | Control-Neg | 0.1856 | 0.157 | 7188 | 1.181 | 0.4644 |
| Low | Pos-Neg | 0.2119 | 0.157 | 7503 | 1.351 | 0.3673 |

| **EPN. Left parietal cluster** | |  |  |  |  |  |
| --- | --- | --- | --- | --- | --- | --- |
| **Valence** | **Contrast** | **Estimate** | **SE** | **df** | **t-value** | **p** |
| Neutral | Control-Pos | -0.372 | 0.167 | 7537 | -2.235 | 0.0654 |
| Neutral | Control-Neg | -0.662 | 0.170 | 7450 | -3.906 | 0.0003 |
| Neutral | Pos-Neg | -0.290 | 0.170 | 7598 | -1.706 | 0.2030 |
| High | Control-Pos | -0.689 | 0.167 | 7517 | -4.118 | 0.0001 |
| High | Control-Neg | -0.376 | 0.171 | 7515 | -2.199 | 0.0713 |
| High | Pos-Neg | 0.313 | 0.170 | 7613 | 1.839 | 0.1570 |
| Low | Control-Pos | -0.370 | 0.167 | 7539 | -2.215 | 0.0686 |
| Low | Control-Neg | -0.335 | 0.171 | 7447 | -1.963 | 0.1215 |
| Low | Pos-Neg | 0.035 | 0.170 | 7608 | 0.206 | 0.9769 |

**Appendix 6. Linear mixed-effects models on response times data.**

**Table 12.** *Linear Mixed-effects Model for Response Times*

| **Fixed effects** |  |  |  |  | |  | |  |
| --- | --- | --- | --- | --- | --- | --- | --- | --- |
|  | **Estimate** | **SE** | **df** | **t-value** | | **p** | |  |
| Intercept: Valence(Neut), Mood(Ctrl) | 844.88 | 46.76 | 23.70 | 18.069 | | 2.32e-15* | |  |
| Valence (High) | -70.89 | 15.27 | 5802.65 | -4.642 | | 3.53e-06* | |  |
| Valence (Low) | -43.10 | 15.34 | 5802.60 | -2.810 | | 0.0050* | |  |
| Mood (Pos) | -43.22 | 37.42 | 28.70 | -1.155 | | 0.2576 | |  |
| Mood (Neg) | -76.10 | 35.35 | 29.89 | -2.153 | | 0.0395* | |  |
| Valence(High):Mood(Pos) | 22.71 | 21.55 | 5802.68 | 1.054 | | 0.2919 | |  |
| Valence(Low):Mood(Pos) | -22.34 | 21.60 | 5802.14 | -1.034 | | 0.3012 | |  |
| Valence(High):Mood(Neg) | 30.28 | 21.72 | 5802.37 | 1.394 | | 0.1635 | |  |
| Valence(Low):Mood(Neg) | -14.91 | 21.76 | 5802.21 | -0.685 | | 0.4934 | |  |
|  |  |  |  |  | |  | |  |
| **Random effects** |  |  |  |  | |  | |  |
| **Groups** | **Name** | **Variance** | **Std.Dev.** |  | |  | |  |
| Participant | Mood (Ctrl) | 45342 | 212.9 |  | |  | |  |
|  | Mood (Pos) | 25382 | 159.3 |  | |  | |  |
|  | Mood (Neg) | 21939 | 148.1 |  | |  | |  |
| Residual |  | 75054 | 274 |  | |  | |  |
|  |  |  |  |  | |  | |  |
| Model: rt ~ valence * mood + (1 + mood \| participant) | | | | |  | |  | |
| Number of observations: 5864, groups: participant, 22 | | | | |  | |  | |

**Appendix 7. IAPS images used for mood induction**

Positive mood induction: 1340, 1440, 1460, 1500, 1600, 1601, 1620, 1710, 1721, 1750, 1999, 2070, 2080, 2091, 2208, 2209, 2216, 2260, 2310, 2311, 2331, 2340, 2341, 2345, 2360, 2391, 2530, 2550, 2650, 5600, 5621, 5629, 5779, 5780, 5820, 5830, 5831, 5910, 5982, 7325, 7502, 8034, 8080, 8090, 8120, 8162, 8170, 8190, 8200, 8210, 8300, 8370, 8380, 8420, 8461, 8470, 9490, 8497, 8510, 8540

Negative mood induction: 1050, 1220, 1274, 2120, 2141, 2205, 2276, 2691, 2700, 2710, 2750, 2900, 3180, 3181, 3220, 3230, 3300, 3301, 3350, 3530, 3550, 6212, 6213, 6312, 6313, 6830, 6831, 6834, 6836, 6838, 9000, 9102, 9041, 9050, 9140, 9160, 9220, 9250, 9280, 9290, 9330, 9340, 9415, 9421, 9430, 9432, 9470, 9520, 9530, 9560, 9571, 9600, 9611, 9620, 9621, 9622, 9630, 9910, 9911, 9920

Mood reset: 1510, 1540, 1610, 1722, 1850, 2303, 2510, 2655, 4700, 5001, 5201, 5480, 5594, 5849, 5994, 8021, 8180, 8340, 8350, 8496
